# Proteomic Analysis of eIF5B Silencing-Modulated Proteostasis

**DOI:** 10.1371/journal.pone.0168387

**Published:** 2016-12-13

**Authors:** Xu Jiang, Xiaoyong Jiang, Yun Feng, Renhua Xu, Qingtao Wang, Haiteng Deng

**Affiliations:** 1 MOE Key Laboratory of Bioinformatics, School of Life Sciences, Tsinghua University, Beijing, China; 2 School of Nursing, Binzhou Medical University, Yantai, China; 3 Beijing Chaoyang Hospital Affiliated to Capital Medical University, Beijing, China; University of British Columbia, CANADA

## Abstract

Protein translational machinery is an important component of the proteostasis network that maintains cellular proteostasis and regulates aging and other cellular processes. Ample evidence indicates that inhibition of translation initiation factor activities enhances stress resistance in model organisms. Eukaryotic translation initiation factor 5B (eIF5B) acts by joining the pre-40S subunit with the 60S ribosomal unit to form an 80S-like complex during protein translational initiation. Reduced eIF5B expression may disrupt proteostasis and trigger cellular processes associated with stress responses. In this study, the physiological effects of altered eIF5B expression were examined in 293T and HepG2 cells. Cells with eIF5B-knockdown (eIF5B-KN) grew more slowly than control cells, and had a lower level of intracellular reactive oxygen species (ROS), increased resistance to oxidative stress and enhanced autophagy. Proteomic analysis showed that eIF5B knockdown resulted in upregulation of 88 proteins and downregulation of 130 proteins compared with control cells. The differentially expressed proteins were associated with diverse cellular processes including amino acid metabolism, RNA processing and protein metabolism, and DNA synthesis. Autonomous downregulation of the mitogen-activated protein kinase (MAPK) signaling pathway was identified as confirmed by western blotting and qPCR. We proposed that deactivation of MAPK pathway modulated proteostasis and induced prolonged S-phase of the cell-cycle, contributing to the slow growth of eIF5B-KN cells. eIF5B silencing also inactivated the mTOR pathway, downregulated glutamine transporters, enhanced autophagy, and decreased 28S rRNA and 5.8S rRNA expression levels which were reversed by restoration of eIF5B expression. Taken together, these results suggest that eIF5B silencing provides a negative feedback to deactivate MAPK signaling, leading to reduced cell growth. These findings provide a useful resource to further biological exploration of the functions of protein synthesis in regulation of proteostasis and stress responses.

## Introduction

Protein synthesis is a highly regulated cellular process to maintain protein homeostasis. Protein homeostasis changes during aging [1–2], with protein synthesis rates and the activities of translation factors decreasing with age [3–5]. Recent studies in the nematode *Caenorhabditis elegans* found that inhibition of mRNA translation affected longevity, suggesting that protein synthesis-mediated changes in proteostasis are important for regulating the aging process [6–8]. These studies also demonstrated that extension of lifespan and resistance to stress are interconnected and regulated by common signaling pathways, including mammalian target of rapamycin (mTOR), mitogen-activated protein kinase (MAPK), and phosphatidylinositol-3-kinase (PI3K/AKT) [9–10]. Many components of the eukaryotic translation initiation factor (eIF) complex and the ribosome were identified as key players in the regulation of resistance to stress and lifespan extension. These included eIF2B, eIF2G, eIF3F, eIF4A, eIF4G and the ribosomal-protein S6 kinase (S6K) [6, 11–12]. However, little is known about the role of eIF5B in translation-inhibition-mediated proteostasis and cellular processes.

Eukaryotic translation initiation factor 5B (eIF5B) is a GTPase that joins the 40S and 60S ribosomal subunits together, and is essential for translation initiation [13–15]. This factor mediates maturation of the 40S ribosomal subunit and stimulates formation of the 48S initiation complex [16–18], and also participates in cap-independent mRNA translation using internal ribosome entry site (IRES) elements [19–20]. Moreover, upregulation of eIF5B controls cell-cycle arrest and specific developmental stages [21], and eIF5B cleavage has been identified in enterovirus infection [22]. Combined, these studies show that eIF5B is involved in multiple processes associated with mRNA translation.

In this study, eIF5B-mediated changes in proteostasis and signaling pathways were examined to investigate the role of eIF5B in the regulation of proteostasis. We established the eIF5B-knockdown cell lines and comprehensively investigated the effects of eIF5B silencing on cellular processes, protein expression, and cellular responses to oxidative stress.

## Materials and Methods

### Chemicals and reagents

Dulbecco’s modified Eagle medium (DMEM), RPMI-1640 medium, fetal bovine serum (FBS) and penicillin/streptomycin were purchased from Wisent (Saint-Jean-Baptiste, CA). Dithiothreitol (DTT), propidium iodide staining kit and BCA protein assay kit were purchased from Solarbio (Beijing, China). Iodoacetamide (IAA) was purchased from Sigma (St Louis, MO). The TMT labeling reagent was purchased from Thermo-Pierce Biotechnology (Rockford, IL). The Lipofectamine 2000 was purchased from Life Technologies (Waltham, MA). Bafilomycin A1 was purchased from Selleck (Houston TX, USA).

### Establishment of eIF5B knockdown cells using CRISPR/Cas9 technology

To knockdown the eIF5B gene in 293T and HepG2 cells, we used the CRISPR/cas9 system. The eIF5B-specific guide RNA (gRNA) sequence was designed using on-line tool at http://crispr.mit.edu and then cloned into pCas-Guide plasmid (Origene, Rockville, MD). The non-silencing scrambled guide RNA was also cloned into the pCas-Guide plasmid and used as control. The eIF5B-specific gRNA sequence was 5’-GAGCGCCATTGACAAGCAATGGG-3’ and non-silencing scrambled gRNA sequence was 5’-ACGATACAAGGCTGTTAGAGAG-3’. Cells were cultured in a 100 mm dish for 24 hours and transfected with 10 μg of pCas-eIF5B or control plasmids using Lipofectamine 2000 according to the manufacturer’s instruction. After transfection for 3 days, single cells were seeded into 96-well plates using BD FACSAria III Flow Cytometer (Becton Dickinson, Franklin Lakes, NJ). After 2–3 weeks, clones were selected and validated by genotyping, western blotting and Sanger sequencing.

For genotyping, genomic DNA of the clones were extracted from 1 × 10^5^ cells by 10 μl QuickExtract solution according to the manufacturer’s instruction (Epicentre Technologies, Chicago, IL), and 1 μl was used as template of the PCR reaction to amplify the DNA fragment including the gene inactivation locus. The primers used in the PCR reaction were listed as follows: sense strand 5’-CTTGCGCACTGAGAACTCAC-3’ and antisense strand 5’-TTCATCACCACTTTCACCAG-3’. The PCR products were annealed and subjected to T7 endonuclease I cleavage (New England Biolabs, Ipswich, MA). The T7 cleaved and un-cleaved samples were analyzed with a 1.5% agrose gel.

### Detection of cellular reactive oxygen species (ROS)

The ROS in control and eIF5B knockdown cells was detected using CellROX® Deep Red Reagents (Invitrogen, Grand Island, NY) following manufacturer's instructions. Cells were stained with 5 μM CellROX® Deep Red Reagent and incubated at 37°C for 30 minutes. Then cells were washed with PBS and analyzed using BD FACSAriaII Flow Cytometer (Becton Dickinson, Franklin Lakes, NJ).

### Survival rate of eIF5B knockdown cells treated with hydrogen peroxide

Effects of hydrogen peroxide on cell viability were analyzed by trypan blue dye exclusion assay. Briefly, cells were grown to 70% confluency and treated with hydrogen peroxide at different concentrations (200, 400 and 800 μM) for 12 hours. Then cells were harvested and mixed with an equal volume of 0.4% trypan blue solution. After two minutes, the numbers of viable cells (unstained) and nonviable cells (stained) were counted in a hemocytometer. The experiment was carried out in triplicate.

### Cell proliferation assay

Briefly, cells were seeded into each well of 96-well cell culture plates. After cells grew for 0, 12, 24, 36, 48, 60, 72, 84 and 96 hours, the CCK-8 reagent was added and absorbance at 450 nm was measured after incubation with CCK-8 reagent at 37°C for 2 hours. The experiment was repeated three times.

### Sample preparation and quantitative proteomic analysis

About 1x10^7^ cells were lysed using 8 M urea in PBS, and lysate was centrifuged at 14,000 x g for 30 minutes at 4°C. Protein concentrations were determined with the BCA method. Equal amount of proteins from control and eIF5B knockdown cells (100 μg) were reduced with 5 mM dithiothreitol and alkylated with 13 mM iodoacetamide. The concentration of urea was diluted to 1.5 M with PBS. Proteins were digested using sequencing grade trypsin at 37°C overnight and then desalted with Sep-Pak C18 Vac cartridges (Waters, Milford, MA). The eluted peptides were centrifuged with a speedvac until dry, then suspended with 200 mM triethyl ammonium bicarbonate (TEAB). The TMT Label Reagents dissolved in acetonitrile were added into the solutions and incubated for 1 hour at room temperature. Peptides from control cells were labeled by TMT^6^-127 and peptides from eIF5B knockdown cells were labeled by TMT^6^-129. Then 5% hydroxylamine was added and incubated for 15 minutes to quench the reaction. The two samples were mixed and desalted with Sep-Pak C18 Vac cartridge.

As described in detail previously [23], the peptide fractionation was performed on an Ultimate 3000 System. For LC-MS/MS analysis, the peptide fractions were separated by a 120-minute gradient elution with a nano-HPLC system (Proxeon, Denmark) which coupled to a Thermo Scientific Q Exactive mass spectrometer. The generated MS/MS spectra were searched against the human.fasta database downloaded from Uniprot (release date of January 10, 2015; 89105 entries) using a Sequest HT Algorithm of Proteome Discoverer software (version 1.4, Thermo Scientific, USA). The search criteria were set as previously described [23]. Proteins with different expression levels were further confirmed using qPCR or western blotting. The proteomics data have been deposited to the ProteomeXchange Consortium via the PRIDE partner repository with the dataset identifier PXD003111.

### Western blotting

Cells were lysed with RIPA lysis buffer on ice. The western blotting assays were performed as previously described [24]. Antibodies against eIF5B, MEK1 and MEK3 were purchased from Proteintech (Wuhan, China). eIF5 antibody was purchased from Abcam (Cambrdge, MA). Antibodies against EEF1A2, mTOR, phospho-mTOR (Ser2448), P70S6K and phospho-P70S6K (Thr389) were purchased from Sigma (St Louis, MO). Antibodies against Erk1/2, phospho-Erk1/2 (Thr202/Tyr204), p38, phospho-p38 (Thr180/Tyr182) and p62 were purchased from Cell Signaling Technology (Boston, MA). LC3 antibody was purchased from MBL (Nagoya, Japan). β-Actin antibody was purchased from Abmart (Shanghai, China).

### RNA isolation and Real-Time Quantitative PCR (qPCR)

Control and eIF5B knockdown cells were harvested and total RNA was isolated by RNA Isolation System. cDNA was synthesized from 1 μg total RNA with the Reverse Transcription kit. All qPCR reactions were performed as previously described [24]. The primers were either from Primer Bank (http://pga.mgh.harvard.edu/primerbank/) or designed using the Primer Premier 5 software. The primers are listed in S1 Table.

### Cell cycle analysis

About 1x10^6^ cells were seeded into a 6-well culture plate. After 24 hours growing, cells were harvested, washed with ice-cold PBS, and fixed with pre-cooled 70% ethanol for 2 hours at 4°C. Then cells were washed with ice-cold PBS for three times and re-suspended with 100 μl PBS. Cells were incubated with 250 μg/ml RNase A for 30 minutes at 37°C and then 100 μg/ml propidium iodide (PI) in dark for 30 minutes at 4°C. Flow cytometry BD FACSCalibur and the CELLQuest software (Becton Dickinson, NJ) were used for data acquisition and analysis respectively.

### Metabolomics analysis

Briefly, 1x10^6^ cells were seeded into a 6 mm dish and cultured for 24 hours. Equal amount of control and eIF5B knockdown cells were metabolically quenched with 80% pre-cold methanol (-80°C) and harvested by centrifuging at 900 x g for 5 minutes at 4°C. The supernatant containing metabolites was transferred to a fresh tube. The pellets were suspended with 1 ml 80% cold methanol, frozen in liquid nitrogen for 8 minutes, thawed at room temperature, and centrifuged at 3,500 x g at 4°C for 10 minutes. The supernatant was transferred to a fresh tube. Metabolites were extracted by repeating this freeze-thaw cycle for three times. Then, all supernatants were centrifuged at 17,000 x g at 4°C for 15 minutes. The extracted metabolites were dried and analyzed by LC-MS/MS.

For LC-MS/MS analysis, metabolites were separated by a 20-minute gradient elution with a Thermo Scientific Dionex Ultimate 3000 UPLC system which coupled to a Thermo Q Exactive mass spectrometer. The analytical column was ACQUITY UPLCBEH Amide column (1.7 μm, 2.1 x 100 mm; Waters, Mildford, MA). Mobile phase A was consisted of 100% H_2_O and 5 mM ammonium formate, and mobile phase B was consisted of 95% acetonitrile, 5% H_2_O and 5 mM ammonium formate. The data were collected in a data-dependent acquisition mode with mass range 70–1000 m/z using the Xcalibur 2.1.2 software. The full scan and fragment spectra were collected with resolution of 70,000 and 17,500 respectively.

### Statistical method

Statistical analysis was performed using two-tailed Student’s t test in GraphPad Prism 5.0 software. The *P* values less than 0.05 were considered statistically significant.

## Results

### Characterization of eIF5B knockdown cells

**Fig 1:**
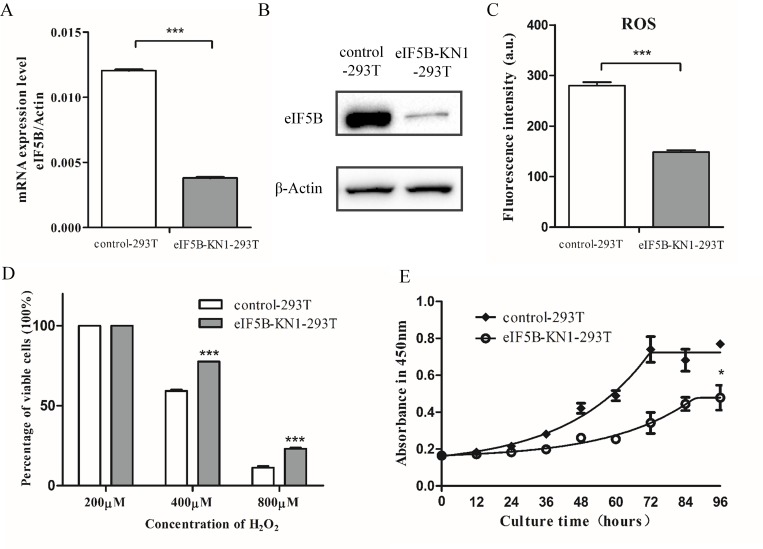
Characterization of eIF5B-KN1-293T and control cells. (A) Semi-quantitative RT-PCR analysis of eIF5B mRNA levels; (B) Western blot analysis of eIF5B protein expression; (C) Cellular ROS levels; (D) Survival rates of cells treated with different concentrations of H_2_O_2_ for 12 h, as determined by trypan blue dye exclusion assay; and (E) Cell growth curves. Data are presented as the mean and standard deviation (*p<0.05; **p<0.01; ***p<0.001; n = 3).

To characterize eIF5B-mediated changes in proteostasis, the CRISPR/Cas9 method was used to knockdown the eIF5B gene in 293T cells. An eIF5B-specific guide RNA (gRNA) sequence was designed to target the start codon of the eIF5B gene. Cells transfected with a plasmid containing non-silencing scrambled guide RNA were used as the control. The eIF5B-KN1-293T cells were heterozygous for a deletion of eleven nucleotides, including the start codon of eIF5B, as determined by Sanger sequencing (S1 Fig). Expression of eIF5B in eIF5B-KN1-293T cells was examined using quantitative PCR (qPCR) and western blotting (Fig 1A and 1B), and confirmed that eIF5B expression in eIF5B-KN1-293T cells was lower than that of the control cells.

Cellular reactive oxygen species (ROS) levels were detected using CellROX® Deep Red Reagents. The eIF5B-KN1-293T cells exhibited much weaker fluorescence than the control cells, indicating that the ROS levels in eIF5B-KN1-293T cells were half of that of controls (Fig 1C). To determine the susceptibility of eIF5B-KN1-293T cells to oxidative stress, cells were treated with different concentrations of H_2_O_2_ for 12 h, and the percentage of viable cells were determined by trypan blue dye exclusion assay (Fig 1D). When cells were treated with 800 μM H_2_O_2_ for 12 h, the percentages of viable cells were 10% and 25% for the control and eIF5B-KN1-293T cells, respectively (Fig 1D). This result indicates that eIF5B-KN1-293T cells are more resistant to H_2_O_2_ treatment. Reduced eIF5B expression in eIF5B-KN1-293T cells also corresponded with a reduction in cell proliferation rates in comparison with control cells (Fig 1E).

To examine whether eIF5B knockdown induced similar changes in other cell lines, we also knocked down eIF5B expression in HepG2 cells using the CRISPR/cas9 method. Expression level of eIF5B in HepG2 knockdown cells was approximately 50% of that of the control cells (S2A and S2B Fig). Consistent with the results obtained in the 293T knockdowns, eIF5B knockdown in HepG2 cells decreased cellular ROS and increased cell resistance to oxidative stress (S2C and S2D Fig). In cells treated with 800 μM H_2_O_2_ for 12 h, the percentages of viable cells were 20% and 30% for the control and eIF5B-knockdown cells, respectively (S2D Fig). The cell proliferation rate was also decreased in the HepG2 eIF5B knockdown cell line (S2E Fig). Together, these results show that eIF5B silencing induces phenotypical changes that correlate with enhanced resistance to stress.

### Quantitative proteomic analysis of eIF5B knockdown and control cells

**Fig 2:**
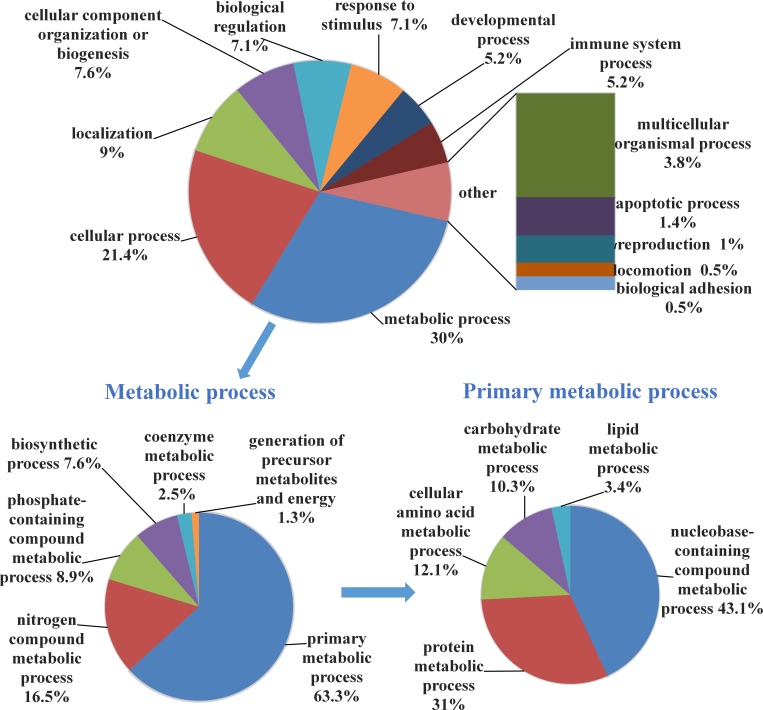
Functional classification of proteins downregulated in eIF5B knockdown cells.

Next, quantitative proteomic analysis was carried out on eIF5B-KN1-293T and control cell lines. Equal amounts of total protein isolated from eIF5B-KN1-293T and control cells were trypsin digested and labeled with TMT reagents. Peptides were fractionated using an off-line HPLC system, and the fractions were further analyzed by nano-LC-MS/MS. A TMT-based quantification method was used to identify and quantify differentially expressed proteins. Experiments were repeated three times and the false-positive rate set to less than 1%. In total, 7516 proteins were identified in each cell line. Using TMT ratios (<0.75 or >1.33), 218 proteins were selected as differentially expressed between eIF5B-KN1-293T and controls, of which 88 were upregulated and 130 downregulated (S2 and S3 Tables). To better appreciate the biological significance of these changes, the Gene Ontology (GO) was used to cluster the proteins according to their molecular functions. The genes encoding each protein are annotated according to their GO functional classifications and presented as a pie plot in Fig 2.

The 130 downregulated proteins were classified into several significant clusters of biological processes, including metabolic processes and cellular processes. Within metabolic processes, more than half of the proteins were associated with primary metabolic processes, including nucleobase-containing compound metabolic process, protein metabolic process, and cellular amino acid metabolic process. Within cellular processes, proteins associated with cytoskeletal reorganization were differentially expressed, including actin-related protein 2/3 complex subunit 1A, angiomotin, coactosin-like protein, and fascin. These protein changes are consistent with changes in cell morphology that were observed in our study. Indeed, eIF5B-KN1-293T cells had fewer membrane protrusions and more cell-to-cell contacts than control cells (S3 Fig). Together, these data suggest that eIF5B knockdown mainly affects cell metabolism and cell morphology.

Among the 88 upregulated proteins, eIF5 is a GTPase-activating eukaryotic translation initiation factor that catalyzes the hydrolysis of eIF2-bound GTP following 43S pre-initiation complex binding to mRNA. Upregulation of eIF5 may compensate for the reduction of eIF5B in eIF5B-KN1-293T cells by promoting 48S initiation complex formation. On the other hand, elongation factor 1-alpha 2 (EEF1A2) was downregulated in response to eIF5B knockdown in eIF5B-KN1-293T cells. The changes in eIF5 and EEF1A2 expression were confirmed by western blotting (S4 Fig).

### MAPK signaling pathways were downregulated in eIF5B knockdown cells

**Fig 3:**
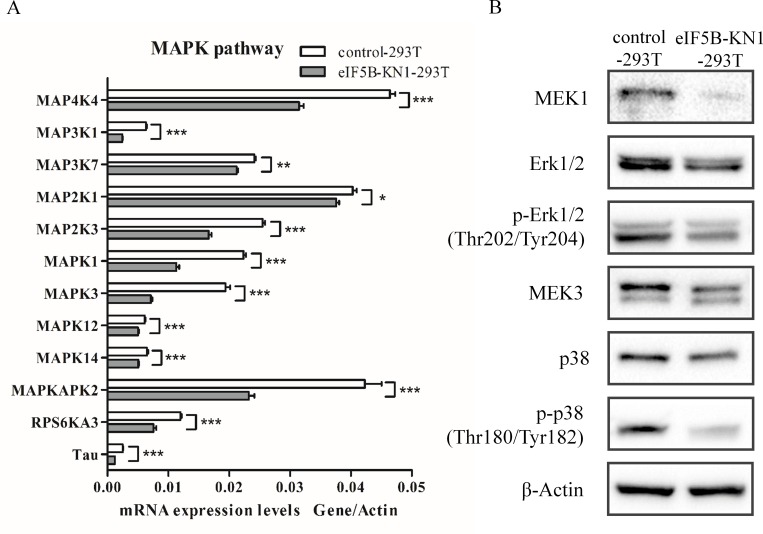
Quantitative PCR and western blot analysis of the eIF5B-knockdown induced deactivation of MAPK signaling pathways. (A) Quantitative PCR analysis of mRNA expression of selected genes in MAPK signaling pathways (*p<0.05; **p<0.01; ***p<0.001; n = 3); and (B) Total cell lysates immunoblotted with MEK1, Erk1/2, phospho-Erk1/2 (Thr202/Tyr204), MEK3, p38 and phospho-p38 (Thr180/Tyr182) antibodies.

Strikingly, proteins associated with the MAPK signaling pathway were downregulated in eIF5B-KN1-293T cells, including isoform 1B of beta-arrestin-1 (ARRB1), isoform tau-A of microtubule-associated protein tau, MAPK-activated protein kinase 2 (MAPKAPK2), ribosomal protein S6 kinase alpha-3 (RPS6KA3), MAPK1, MAPK3, MAPK12 and MAPK14. Quantitative PCR analysis further confirmed this finding; mRNA levels of 12 genes within the MAPK signaling pathway were downregulated, including two MAPK kinase kinases (MAPKKKs), two MAPK kinases (MAPKKs) and four MAPKs (Fig 3A). The downregulation of kinases in the MAPK signaling pathway reduced substrate phosphorylation and suppressed MAPK activity, as determined by western blotting. MAPK1 (also known as Erk2) and MAPK3 (also known as Erk1) are phosphorylated and activated by the dual-specificity protein kinase MAP2K1 (also known as MEK1). Western blotting revealed that MEK1 expression was downregulated in eIF5B knockdown cells, leading to reduced Erk1/2 phosphorylation (Fig 3B and S5 Fig). MAPK12 and MAPK14, two isoforms of p38, are phosphorylated and activated by the dual-specificity protein kinase MAP2K3 (also known as MEK3). The eIF5B knockdown induced downregulation of MEK3 and reduced p38 phosphorylation, as confirmed by western blotting in both the eIF5B-KN1-293T and eIF5B-KN1-HepG2 cell lines (Fig 3B and S5 Fig). Combined, these results demonstrate that eIF5B silencing induced autonomous downregulation of the Erk1/2 and p38 MAPK signaling pathways.

### Downregulation of eIF5B led to a prolonged S-phase of cell cycle in eIF5B knockdown cells

**Fig 4:**
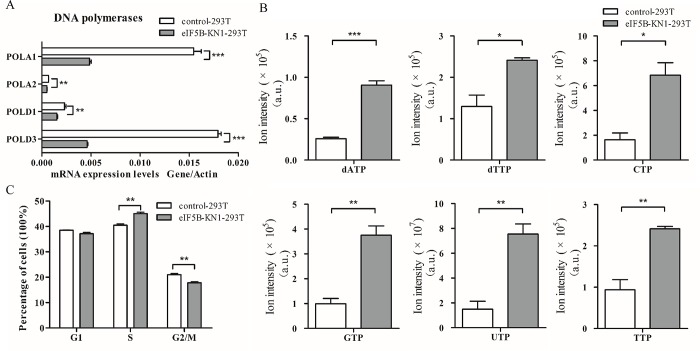
Prolonged S-phase of cell cycle in eIF5B-knockdown cells. (A) Quantitative PCR analysis of mRNA expression levels of DNA polymerases in the DNA synthesis pathway; (B) Nucleotides accumulated within cells; and (C) Cell numbers in each cell cycle phase as determined by flow cytometry (*p<0.05; **p<0.01; ***p<0.001; n = 3).

It is known that MAPK signal pathways regulate expression of DNA polymerases [25–27]. As expected, four DNA polymerases were downregulated among the differentially expressed proteins in eIF5B-KN1-293T cells (S2 and S3 Tables), including DNA polymerases alpha subunits POLA1 and POLA2, and DNA polymerase catalytic subunits POLD1 and POLD3. These results were confirmed with qPCR (Fig 4A). Downregulation of DNA polymerases led to an accumulation of cellular deoxyadenosine triphosphate (dATP) and deoxythymidine triphosphate (dTTP), as well as nucleotide triphosphates GTP, CTP, UTP, and TTP, as determined by metabolomic analysis (Fig 4B). Furthermore, a decrease in DNA polymerases was known to interfere cell cycle. Indeed, cell cycle analysis of eIF5B-KN and control cells showed that eIF5B knockdown in both 293T and HepG2 cells had higher S-phase accumulation (Fig 4C and S6 Fig). These results suggest that the deactivation of MAPK signal pathway downregulates DNA polymerases expression and prolongs the S phase of cell cycle, contributing to the decreased cell proliferation (Fig 1E and S2E Fig).

### eIF5B silencing enhanced autophagy and downregulated rRNA expression in eIF5B knockdown cells

**Fig 5:**
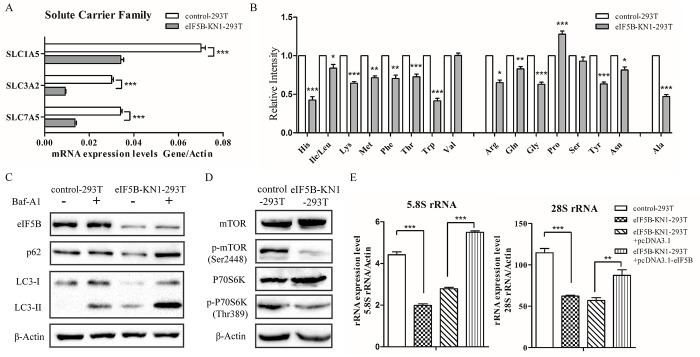
Amino acid accumulation and autophagy induced in eIF5B knockdown cells. (A) Quantitative PCR analysis of mRNA expression of essential amino acid transporters; (B) Levels of intracellular amino acids in eIF5B knockdown cells compared with control cells; (C) Protein expression levels of LC3 and p62 following cell culture for 2 h in the presence or absence of 100 nM Baf-A1, as determined by western blotting; (D) Total cell lysates immunoblotted with mTOR, phospho-mTOR (Ser2448), P70S6K and phospho-P70S6K (Thr389) antibodies; (E) RT-PCR analysis of rRNA concentrations using rRNA-specific primers listed in S1 Table (*p<0.05; **p<0.01; ***p<0.001; n = 3).

The eIF5B knockdown also induced other changes in cellular processes. Proteomic analysis showed that expression levels of three amino acid transporters, 4F2 cell-surface antigen heavy chain (SLC3A2), neutral amino acid transporter (SLC1A5) and large neutral amino acids transporter small subunit 1 (SLC7A5) were all downregulated in the eIF5B-KN1-293T cell line. These results were confirmed by qPCR (Fig 5A), and were consistent with metabolomic analysis that found reduced glutamine and other amino acid levels in eIF5B-KN1-293T cells (Fig 5B). Furthermore, to detect autophagy flux, cells were cultured in the presence or absence of bafilomycin A1 (Baf-A1) and changes in LC3 and p62 levels were determined by western blotting. The lysosomal inhibitor Baf-A1 blocks autophagy by preventing fusion of autophagosomes with lysosomes. Knockdown of eIF5B in 293T cells increased LC3-II and reduced p62 protein expression, indicating that autophagy was activated in the eIF5B-KN1-293T cell line. This result was further confirmed by the increased accumulation of LC3-II and p62 in eIF5B-KN1-293T cells following treatment with Baf-A1 (Fig 5C). The eIF5B knockdown also enhanced autophagy formation in HepG2 cells, as determined by autophagy flux measurements (S7 Fig).

Inhibition of the mTOR/P70S6K pathway has also been shown to induce autophagy [28–29]. To examine whether eIF5B-knockdown induced autophagy through inhibition of the mTOR pathway, we measured mTOR and P70S6K phosphorylation levels in control and eIF5B-KN cells using western blot analysis (Fig 5D). Both mTOR and P70S6K phosphorylation were reduced in eIF5B knockdown cells.

Previous studies showed that the mTOR pathway regulated ribosome biogenesis [30]. In the present study, total RNA was extracted from each of eIF5B-KN1-293T and control cells and analyzed by real time quantitative reverse transcriptase PCR (Fig 5E). Expression levels of 28S and 5.8S rRNAs were higher in the control in comparison with the eIF5B-KN1-293T cell line. The 28S and 5.8S rRNAs are associated with the 60S ribosomal subunit and are encoded by a single pre-rRNA transcription unit. These results suggest that eIF5B knockdown affects expression levels of 28S and 5.8S rRNAs. Furthermore, overexpression of eIF5B in the eIF5B-KN1-293T cells increased both 28S and 5.8S rRNA expression levels.

## Discussion

Protein synthesis machinery is the key component of the proteostasis network. Previous studies have revealed that protein synthesis activities decline with aging. Moreover, attenuation of mRNA translation increases stress responses and extends the lifespan of model organisms. Attenuation of protein synthesis can be achieved by knockdown of ribosome subunits and translation initiation factors such as eIF1, eIF2, and eIF4 subunits [31]. However, the effects of eIF5B knockdown on cellular processes have not previously been reported, even though it is a major subunit of the protein synthesis machinery.

In the present study, 293T and HepG2 cells with reduced eIF5B expression were established and characterized. Reduced eIF5B expression suppressed cell growth and proliferation, decreased cellular ROS levels, and increased resistance to oxidative stress. To rule out the off-target and non-specific effects of the eIF5B-specific guide RNA (sgRNA), we repeated all the experiments with a different guide RNA sequence as listed in (S1 Table). Analysis of the stable cell line in which eIF5B was knocked down with the second sgRNA generated almost identical results in cell proliferation, ROS level, prolonged S phase of cell cycle and enhanced autophagy (S8 and S9 Figs) as reported for cells derived from the first sgRNA oligonucleotide. Furthermore, similar effects of eIF5B silencing on cellular processes were observed in both embryonic kidney cells and liver carcinoma cells in which eIF5B was knocked down. These findings suggest that eIF5B is also a factor that contributes to the cell proliferation and stress responses.

Quantitative proteomics comparing eIF5B-KN cells against the control background identified differential expression of proteins associated with DNA synthesis, protein metabolism, and amino acid metabolism. Importantly, proteins associated with the MAPK-signaling pathway were downregulated, including four MAPKs, two MAPKKs and two MAPKKKs. MAPK signaling is essential to cell growth and proliferation [32]. Downregulation of MAPK proteins suppresses the activity of the MAPK signaling pathway. This process further contributes to the slow growth of eIF5B-KN cells, indicating that eIF5B knockdown provides a negative feedback to regulation of cell proliferation. Transcription factors, such as YB-1, Myc, and E2F, that regulate expression of DNA polymerases are known downstream targets of the MAPK pathway [25–27]. Indeed, in our study, expression levels of multiple DNA polymerases were downregulated in the eIF5B-KN cells, resulting in the accumulation of nucleotides. Our results also show that eIF5B-KN cells have prolonged S-phase of cell cycle, consistent with previous studies that have shown the involvement of the MAPK pathway in cell cycle regulation [33–34].

Metabolomic analysis revealed that cellular amino acids levels were decreased in eIF5B-KN cells, which was attributable to downregulation of the glutamine transporters SLC1A5, SLC3A2, and SLC7A5. Previous studies have shown that expression levels of these transporters are regulated by the MAPK signaling pathway [35–36], and downregulation of SLC1A5 enhances autophagy [37–38]. Enhanced autophagy was observed in eIF5B-KN cells in the present study. Moreover, eIF5B silencing inactivated mTOR signal pathway, which regulated both autophagy and ribosome biogenesis, leading to enhanced autophagy and a decrease in 28S and 5.8S rRNA expression levels [29, 30]. An interesting result observed was that eIF5B knockdown enhanced stress responses. Although the underlying mechanism is still unclear, our results suggest that enhanced autophagy and slow growth contribute to the low level ROS and enhanced resistance to oxidative stress in eIF5B-KN cells [39].

Taken together, the results of our study demonstrate that eIF5B knockdown alters proteostasis and enhances autophagy, implicating eIF5B as a potential target for manipulating the stress-response process. Our data also highlight the strength of proteomics as a powerful approach to deciphering the complex cellular processes associated with protein translation.

## Supporting Information

S1 Fig Sanger sequencing of the eIF5B gene in the control and eIF5B-KN1-293T cells. The 11nt deletion was detected and marked by the red line, and the start codon was labeled by a red box.
(DOCX)

S2 Fig Characterization of eIF5B-KN1-HepG2 and control cells. (A) Semi-quantitative RT-PCR analysis of eIF5B mRNA level; (B) Western blot analysis of eIF5B protein expression; (C) Cellular ROS levels; (D) Survival rates of cells treated with different concentrations of H_2_O_2_ for 12 h, as determined by trypan blue dye exclusion assay; and (E) Cell growth curves. Data are presented as the mean and standard deviation (*p<0.05; **p<0.01; ***p<0.001; n = 3).
(DOCX)

S3 Fig Morphologies of the control and eIF5B-KN1-293T cells. (DOCX)

S4 Fig Western blotting of eIF5 and EEF1A2 in the eIF5B-KN1-293T and control cells. (DOCX)

S5 Fig Western blotting analysis of the eIF5B-knockdown induced deactivation of MAPK signaling pathways in HepG2 cells. (DOCX)

S6 Fig Cell cycle of eIF5B-KN1-HepG2 and control cells. (DOCX)

S7 Fig Detection of autophagy flux in eIF5B-KN1-HepG2 and control cells. Cells were cultured for 2 hours in the presence or absence of 100 nM Baf-A1 and protein expression levels of LC3 and p62 were determined by western blotting.
(DOCX)

S8 Fig Characterization of control and eIF5B-KN2-293T cells, in which eIF5B was knocked down with the second sgRNA oligonucleotide. (A) Semi-quantitative RT-PCR analysis of eIF5B mRNA level; (B) Western blot analysis of eIF5B protein expression; (C) Cellular ROS levels; (D) Survival rates of cells treated with different concentrations of H_2_O_2_ for 12 h, as determined by trypan blue dye exclusion assay; (E) Cell growth curves; (F) Western blot analysis of the eIF5B-knockdown induced deactivation of MAPK signaling pathways; (G) Cell numbers in each cell cycle phase as determined by flow cytometry; and (H) Detection of autophagy flux. Data are presented as the mean and standard deviation (*p<0.05; **p<0.01; ***p<0.001; n = 3).
(DOCX)

S9 Fig Characterization of control and eIF5B-KN2-HepG2 cells, in which eIF5B was knocked down with the second sgRNA oligonucleotide. (A) Semi-quantitative RT-PCR analysis of eIF5B mRNA level; (B) Western blot analysis of eIF5B protein expression; (C) Cellular ROS levels; (D) Survival rates of cells treated with different concentrations of H_2_O_2_ for 12 h, as determined by trypan blue dye exclusion assay; (E) Cell growth curves; (F) Western blot analysis of the eIF5B-knockdown induced deactivation of MAPK signaling pathways; (G) Cell numbers in each cell cycle phase as determined by flow cytometry; and (H) Detection of autophagy flux. Data are presented as the mean and standard deviation (*p<0.05; **p<0.01; ***p<0.001; n = 3).
(DOCX)

S1 Table The list of primers used for RT-PCR analysis and eIF5B-specific gRNA sequence. (DOCX)

S2 Table The list of up-regulated proteins in eIF5B-KN1-293T cells compared to the control cells. (DOCX)

S3 Table The list of down-regulated proteins in eIF5B-KN1-293T cells compared to the control cells. (DOCX)
