# Supplementary material for: Proteomic Analysis of eIF5B Silencing-Modulated Proteostasis
Source: PLoS One. 2016 Dec 13;11(12):e0168387. doi: 10.1371/journal.pone.0168387 (PMC5154608; doi:10.1371/journal.pone.0168387)
Supplement: S1 Fig — The 11nt deletion was detected and marked by the red line, and the start codon was labeled by a red box. (DOCX) [file pone.0168387.s001.docx]

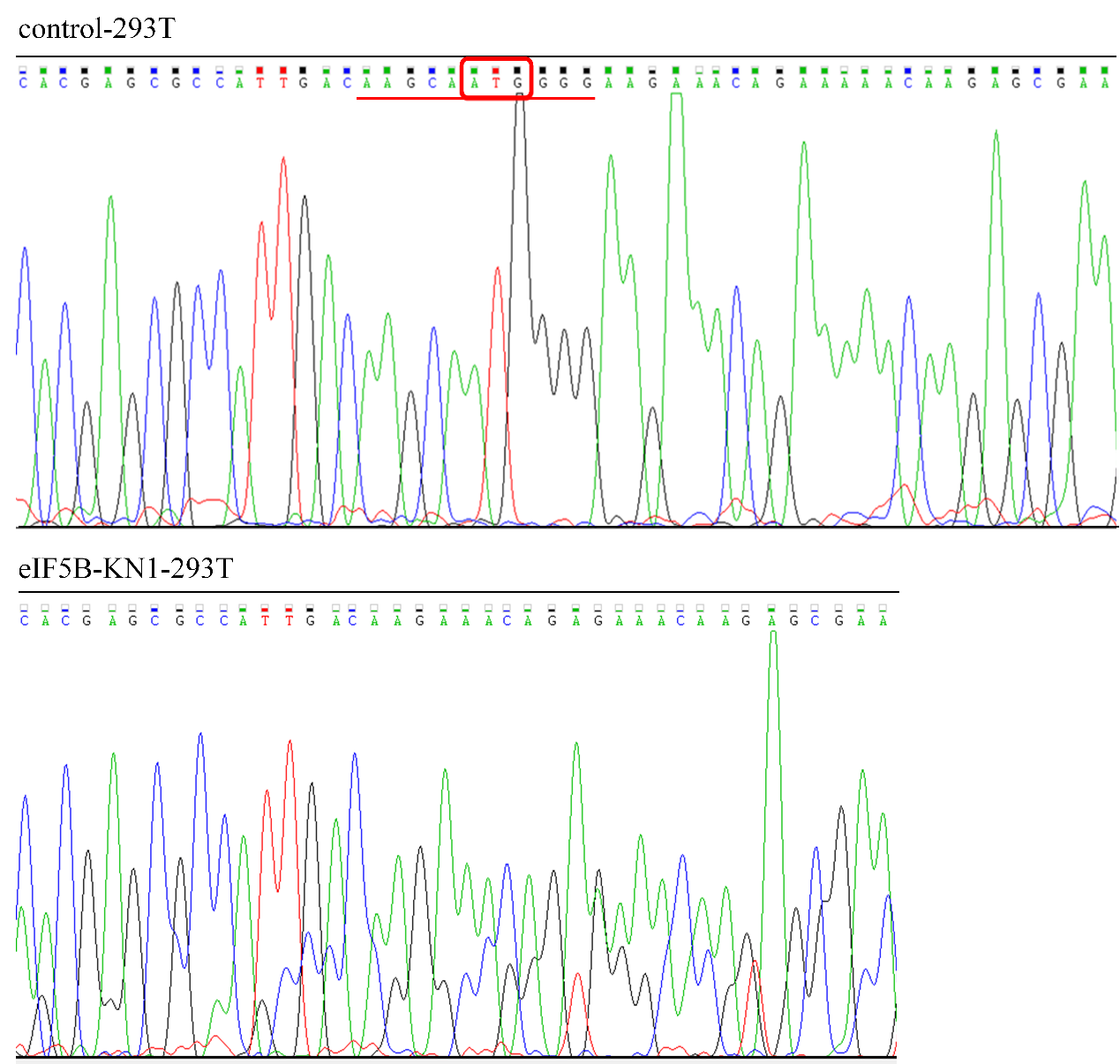


**S1 Fig.** **Sanger sequencing of the eIF5B gene in the control and eIF5B-KN1-293T cells.** The 11nt deletion was detected and marked by the red line, and the start codon was labeled by a red box.
