# Supplementary material for: Proteomic Analysis of eIF5B Silencing-Modulated Proteostasis
Source: PLoS One. 2016 Dec 13;11(12):e0168387. doi: 10.1371/journal.pone.0168387 (PMC5154608; doi:10.1371/journal.pone.0168387)
Supplement: S2 Fig — (A) Semi-quantitative RT-PCR analysis of eIF5B mRNA level; (B) Western blot analysis of eIF5B protein expression; (C) Cellular ROS levels; (D) Survival rates of cells treated with different concentrations of H2O2 for 12 h, as determined by trypan blue dye exclusion assay; and (E) Cell growth curves. Data are presented as the mean and standard deviation (*p<0.05; **p<0.01; ***p<0.001; n = 3). (DOCX) [file pone.0168387.s002.docx]

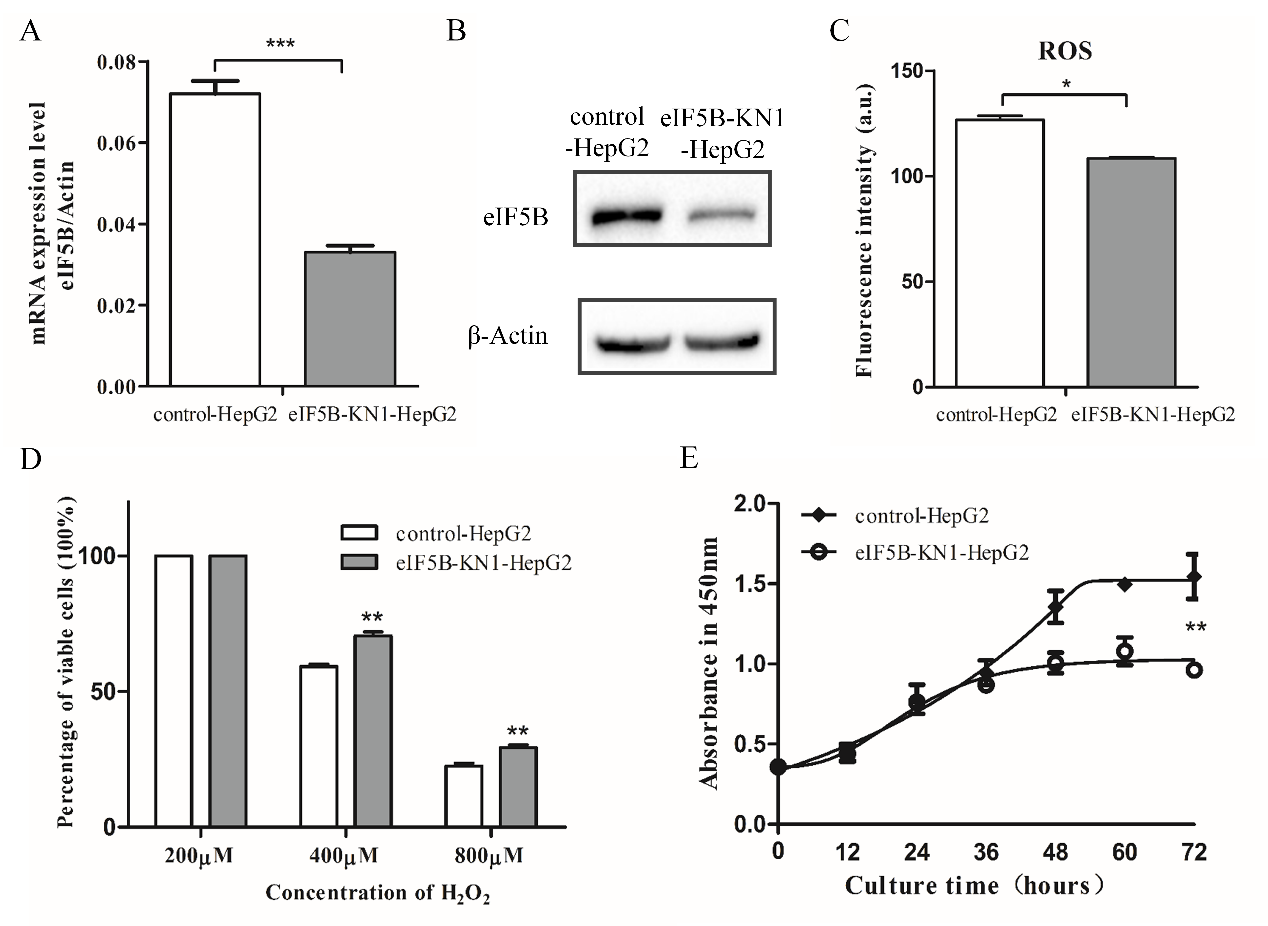


**S2 Fig. Characterization of eIF5B-KN1-HepG2 and control cells.** (A) Semi-quantitative RT-PCR analysis of eIF5B mRNA level; (B) Western blot analysis of eIF5B protein expression; (C) Cellular ROS levels; (D) Survival rates of cells treated with different concentrations of H_2_O_2_ for 12 h, as determined by trypan blue dye exclusion assay; and (E) Cell growth curves. Data are presented as the mean and standard deviation (**p*<0.05; ***p*<0.01; ****p*<0.001; n=3).
