# Supplementary figures and images for: Proteomic Analysis of eIF5B Silencing-Modulated Proteostasis
Source: PLoS One. 2016 Dec 13;11(12):e0168387. doi: 10.1371/journal.pone.0168387 (PMC5154608; doi:10.1371/journal.pone.0168387)

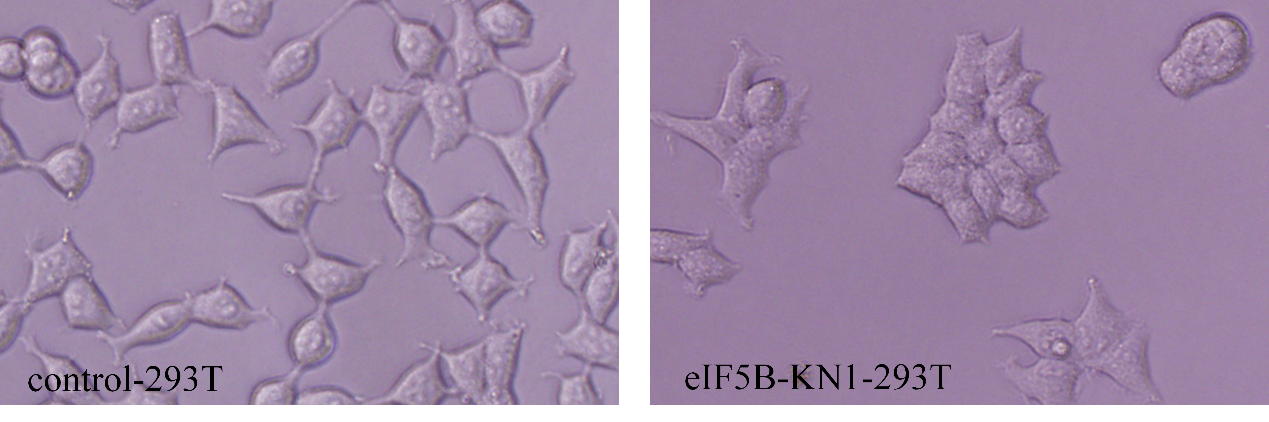


**S3 Fig. Morphologies of the control and eIF5B-KN1-293T cells.**

Supplement: S3 Fig — (DOCX) [file pone.0168387.s003.docx]

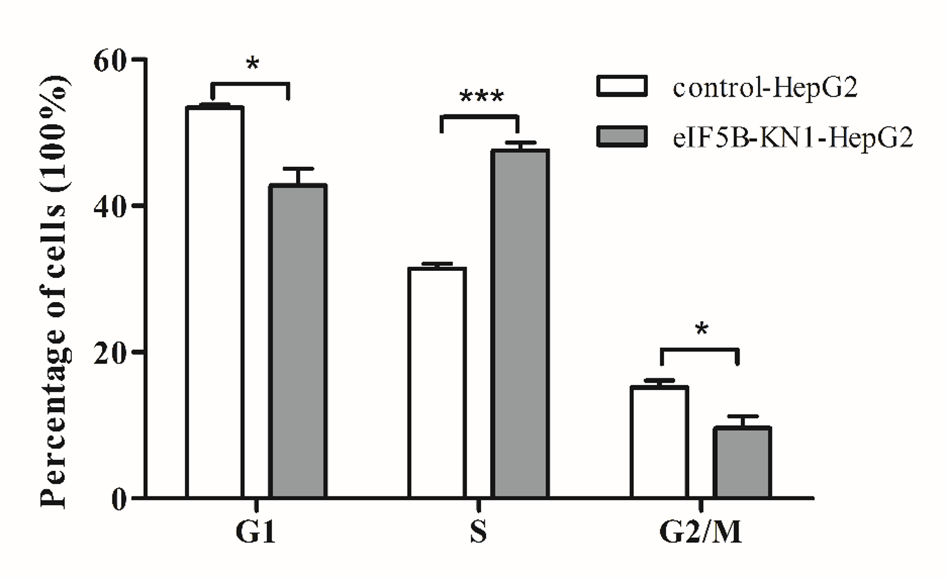


**S6 Fig. Cell cycle of eIF5B-KN1-HepG2 and control cells.**

Supplement: S6 Fig — (DOCX) [file pone.0168387.s006.docx]
