# Supplementary material for: Proteomic Analysis of eIF5B Silencing-Modulated Proteostasis
Source: PLoS One. 2016 Dec 13;11(12):e0168387. doi: 10.1371/journal.pone.0168387 (PMC5154608; doi:10.1371/journal.pone.0168387)
Supplement: S4 Fig — (DOCX) [file pone.0168387.s004.docx]

**S4 Fig. Western blotting of eIF5 and EEF1A2 in the eIF5B-KN1-293T and control cells.**
