# Supplementary material for: Proteomic Analysis of eIF5B Silencing-Modulated Proteostasis
Source: PLoS One. 2016 Dec 13;11(12):e0168387. doi: 10.1371/journal.pone.0168387 (PMC5154608; doi:10.1371/journal.pone.0168387)
Supplement: S5 Fig — (DOCX) [file pone.0168387.s005.docx]

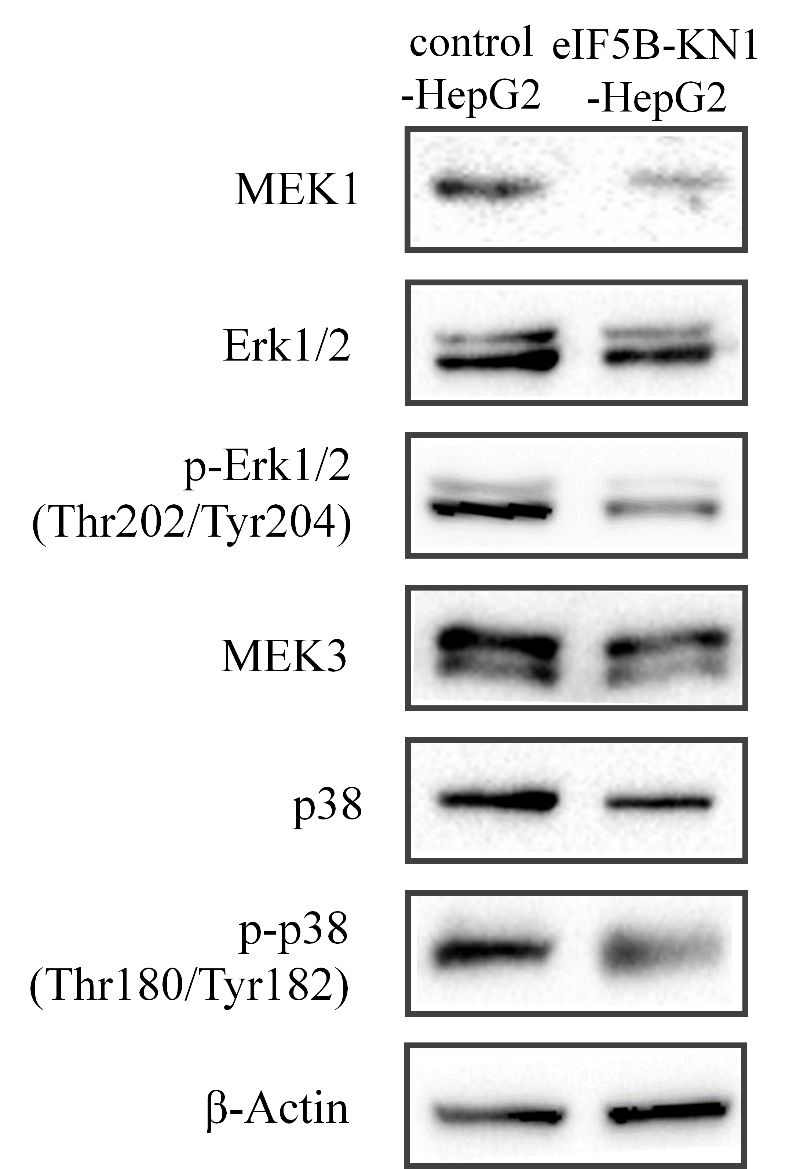


**S5 Fig. Western blotting analysis of the eIF5B-knockdown induced deactivation of MAPK signaling pathways in HepG2 cells.**
