# Supplementary material for: Proteomic Analysis of eIF5B Silencing-Modulated Proteostasis
Source: PLoS One. 2016 Dec 13;11(12):e0168387. doi: 10.1371/journal.pone.0168387 (PMC5154608; doi:10.1371/journal.pone.0168387)
Supplement: S7 Fig — Cells were cultured for 2 hours in the presence or absence of 100 nM Baf-A1 and protein expression levels of LC3 and p62 were determined by western blotting. (DOCX) [file pone.0168387.s007.docx]

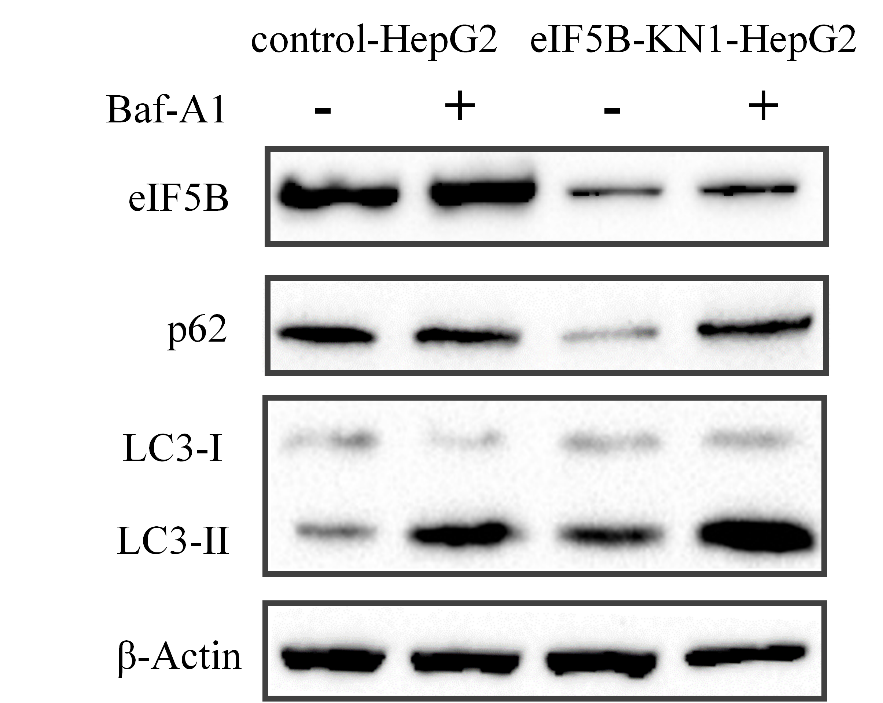


**S7 Fig. Detection of autophagy flux in eIF5B-KN1-HepG2 and control cells.** Cells were cultured for 2 hours in the presence or absence of 100 nM Baf-A1 and protein expression levels of LC3 and p62 were determined by western blotting.
