# Supplementary material for: Proteomic Analysis of eIF5B Silencing-Modulated Proteostasis
Source: PLoS One. 2016 Dec 13;11(12):e0168387. doi: 10.1371/journal.pone.0168387 (PMC5154608; doi:10.1371/journal.pone.0168387)
Supplement: S8 Fig — (A) Semi-quantitative RT-PCR analysis of eIF5B mRNA level; (B) Western blot analysis of eIF5B protein expression; (C) Cellular ROS levels; (D) Survival rates of cells treated with different concentrations of H2O2 for 12 h, as determined by trypan blue dye exclusion assay; (E) Cell growth curves; (F) Western blot analysis of the eIF5B-knockdown induced deactivation of MAPK signaling pathways; (G) Cell numbers in each cell cycle phase as determined by flow cytometry; and (H) Detection of autophagy flux. Data are presented as the mean and standard deviation (*p<0.05; **p<0.01; ***p<0.001; n = 3). (DOCX) [file pone.0168387.s008.docx]

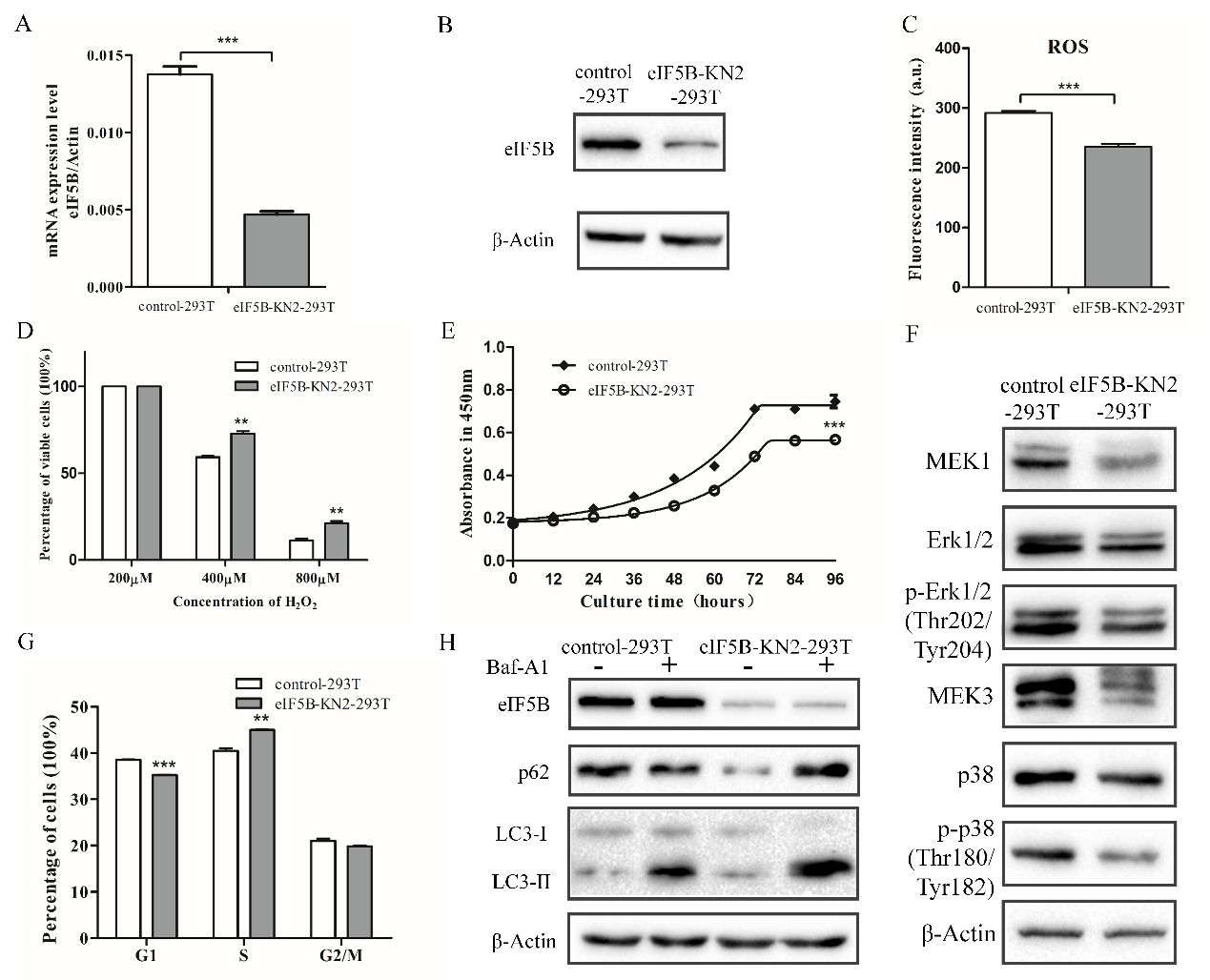


**S8 Fig. Characterization of control and eIF5B-KN2-293T cells, in which eIF5B was knocked down with the second sgRNA oligonucleotide.** (A) Semi-quantitative RT-PCR analysis of eIF5B mRNA level; (B) Western blot analysis of eIF5B protein expression; (C) Cellular ROS levels; (D) Survival rates of cells treated with different concentrations of H_2_O_2_ for 12 h, as determined by trypan blue dye exclusion assay; (E) Cell growth curves; (F) Western blot analysis of the eIF5B-knockdown induced deactivation of MAPK signaling pathways; (G) Cell numbers in each cell cycle phase as determined by flow cytometry; and (H) Detection of autophagy flux. Data are presented as the mean and standard deviation (**p*<0.05; ***p*<0.01; ****p*<0.001; n=3).
