# Supplementary material for: Proteomic Analysis of eIF5B Silencing-Modulated Proteostasis
Source: PLoS One. 2016 Dec 13;11(12):e0168387. doi: 10.1371/journal.pone.0168387 (PMC5154608; doi:10.1371/journal.pone.0168387)
Supplement: S1 Table — (DOCX) [file pone.0168387.s010.docx]

**S1 Table.** **The list of primers used for RT-PCR analysis and eIF5B-specific gRNA sequence.**

| Primer name | Primer sequence |
| --- | --- |
| eIF5B-F | TGAAGGCTTCAGTGATGTTGGA |
| eIF5B-R | AACTCCTAAACTATCAGCCATTTCTTGT |
| MAP4K4-F | CCACCAGTATTGAGCCCAGG |
| MAP4K4-R | TTGTGAGGTGGCCGTACATC |
| MAP3K1-F | TGAACAGCTATGAACGAGGCCAGT |
| MAP3K1-R | TTTCCTGTTCACCTAGGGCCAGTT |
| MAP3K7-F | CGACGAATCATGTGGGCTGTTCA |
| MAP3K7-R | CGTACGAGTCATCAGGCTCTCAA |
| MAP2K1-F | GCCCTCCAACATCCTAGTCA |
| MAP2K1-R | GACAGTCCCATGCTCCAGAT |
| MAP2K3-F | GTGGAGCCCGCAGTCCTCTA |
| MAP2K3-R | GGGTGGCTTGGACATGCAG |
| MAPK1-F | TCTGGCAGGCAGGCAGGCAAT |
| MAPK1-R | TGACCGGGAGGAGGAAGGAAGA |
| MAPK3-F | GGCCCGAAACTACCTACAGT |
| MAPK3-R | TAGGTAGGTCATCCAGCTCCA |
| MAPK12-F | ACATGAAGGGCCTCCCCG |
| MAPK12-R | TCTCCTTGGAGACCCTGG |
| MAPK14-F | GTGCCCGAGCGTTACCAGACC |
| MAPK14-R | CTGTAAGCTTCTGACATTTC |
| MAPKAPK2-F | CAGCAGTTCCCGCAGTTC |
| MAPKAPK2-R | CGAATTTCTCCTGGGTCCTC |
| RPS6KA3-F | GAACGAGAGGCCAGTGCTGT |
| RPS6KA3-R | CGCTCTCAGCTGTTTTGCAA |
| Tau-F | GACAAAAAAGCCAAGGGGGC |
| Tau-R | AGGGACGGGGTGCGGGAGCG |
| POLA1-F | CACTGGAGAAACTTACTACCG |
| POLA1-R | AAGAGTTTGCTCAGATTCAC |
| POLA2-F | TGAAGGATGTCCTCGGC |
| POLA2-R | TCCTGACGACCTGCACA |
| POLD1-F | CAACCTGGTCACTGCCTCAC |
| POLD1-R | GTCCCGCTTCCTCATCCTCT |
| POLD3-F | ATGGCGGACCAGCTTTATCT |
| POLD3-R | GCAACCTTGTGGCAGGAATG |
| SLC1A5-F | CCGCTTCTTCAACTCCTTCAA |
| SLC1A5-R | ACCCACATCCTCCATCTCCA |
| SLC3A2-F | TCTTGATTGCGGGGACTAAC |
| SLC3A2-R | GCCTTGCCTGAGACAAACTC |
| SLC7A5-F | GTCCTGTGTCAGAAGCTCAT |
| SLC7A5-R | GTGATCTACTTTAACTGGCCTCT |
| 5.8S rRNA-F | GTGCGTCGATGAAGAACGC |
| 5.8S rRNA-R | AGTGCGTTCGAAGTGTCGAT |
| 28S rRNA-F | AAGCAGGAGGTGTCAGAAA |
| 28S rRNA-R | GTAAAACTAACCTGTCTCACG |
| Actin-F | CTGGAACGGTGAAGGTGACA |
| Actin-R | AAGGGACTTCCTGTAACAATGCA |
| sgRNA2-F | GATCGGCCTTGGCTGCAGAAATAGAG |
| sgRNA2-R | AAAACTCTATTTCTGCAGCCAAGGCC |
