# Supplementary material for: Proteomic Analysis of eIF5B Silencing-Modulated Proteostasis
Source: PLoS One. 2016 Dec 13;11(12):e0168387. doi: 10.1371/journal.pone.0168387 (PMC5154608; doi:10.1371/journal.pone.0168387)
Supplement: S2 Table — (DOCX) [file pone.0168387.s011.docx]

**S2 Table. The list of up-regulated proteins in eIF5B-KN1-293T cells compared to the control cells.**

| Accession | Description | | | Score | | | | Coverage (%) | | | | | Unique Peptides | | | Peptides | | | | | | PSMs | eIF5B(-)/control | |  |  |
| --- | --- | --- | --- | --- | --- | --- | --- | --- | --- | --- | --- | --- | --- | --- | --- | --- | --- | --- | --- | --- | --- | --- | --- | --- | --- | --- |
| Q9BV57 | 1,2-dihydroxy-3-keto-5-methylthiopentene dioxygenase | | | | 75.94 | | | | 62.01 | | | | 9 | | | 9 | | | 22 | | | | 1.4 | | |  |
| E7EMM4 | Acid ceramidase | | | | 15.92 | | | | 11.62 | | | | 4 | | | 4 | | | 4 | | | | 1.6 | | |  |
| P15121 | Aldose reductase | | | | 342.04 | | | | 71.52 | | | | 17 | | | 18 | | | 90 | | | | 1.4 | | |  |
| H7C485 | AT-rich interactive domain-containing protein 4A (Fragment) | | | | 10.24 | | | | 4.01 | | | | 2 | | | 3 | | | 3 | | | | 1.4 | | |  |
| Q13057 | Bifunctional coenzyme A synthase | | | | 71.38 | | | | 28.72 | | | | 9 | | | 9 | | | 16 | | | | 1.4 | | |  |
| O75828 | Carbonyl reductase [NADPH] 3 | | | | 45.52 | | | | 35.74 | | | | 5 | | | 8 | | | 12 | | | | 1.5 | | |  |
| Q02224 | Centromere-associated protein E | | | | 133.81 | | | | 14.25 | | | | 30 | | | 32 | | | 37 | | | | 1.4 | | |  |
| D6RI11 | Complexin-1 | | | | 11.13 | | | | 31.86 | | | | 2 | | | 2 | | | 3 | | | | 1.8 | | |  |
| Q99829 | Copine-1 | | | | 110.78 | | | | 34.26 | | | | 15 | | | 15 | | | 28 | | | | 1.4 | | |  |
| P12532 | Creatine kinase U-type, mitochondrial | | | | 465.70 | | | | 56.83 | | | | 21 | | | 22 | | | 111 | | | | 1.7 | | |  |
| Q00534 | Cyclin-dependent kinase 6 | | | | 104.47 | | | | 45.71 | | | | 10 | | | 11 | | | 26 | | | | 1.4 | | |  |
| Q00535 | Cyclin-dependent-like kinase 5 | | | | 79.22 | | | | 46.92 | | | | 10 | | | 11 | | | 22 | | | | 1.4 | | |  |
| Q8IYA6 | Cytoskeleton-associated protein 2-like | | | | 18.14 | | | | 7.79 | | | | 4 | | | 4 | | | 5 | | | | 1.3 | | |  |
| Q16854 | Deoxyguanosine kinase, mitochondrial | | | | 17.94 | | | | 16.61 | | | | 2 | | | 2 | | | 3 | | | | 1.4 | | |  |
| Q16555 | Dihydropyrimidinase-related protein 2 | | | | 213.73 | | | | 61.71 | | | | 20 | | | 23 | | | 51 | | | | 1.4 | | |  |
| O14531 | Dihydropyrimidinase-related protein 4 | | | | 83.79 | | | | 34.44 | | | | 14 | | | 14 | | | 21 | | | | 1.7 | | |  |
| P25685 | DnaJ homolog subfamily B member 1 | | | | 176.54 | | | | 46.18 | | | | 17 | | | 18 | | | 50 | | | | 1.4 | | |  |
| Q8WXX5 | DnaJ homolog subfamily C member 9 | | | | 154.27 | | | | 55.38 | | | | 17 | | | 17 | | | 39 | | | | 1.4 | | |  |
| O94822 | E3 ubiquitin-protein ligase listerin | | | | 76.21 | | | | 12.06 | | | | 15 | | | 15 | | | 19 | | | | 1.4 | | |  |
| P30040 | Endoplasmic reticulum resident protein 29 | | | | 219.25 | | | | 52.11 | | | | 15 | | | 15 | | | 58 | | | | 1.4 | | |  |
| P55010 | Eukaryotic translation initiation factor 5 | | | | 421.55 | | | | 55.68 | | | | 29 | | | 29 | | | 106 | | | | 1.5 | | |  |
| K7ELL7 | Glucosidase 2 subunit beta | | | | 319.37 | | | | 48.04 | | | | 27 | | | 27 | | | 77 | | | | 1.3 | | |  |
| Q9UIJ7 | GTP:AMP phosphotransferase AK3, mitochondrial | | | | 95.45 | | | | 69.60 | | | | 15 | | | 15 | | | 27 | | | | 1.4 | | |  |
| P48723 | Heat shock 70 kDa protein 13 | | | | 34.35 | | | | 14.44 | | | | 7 | | | 7 | | | 10 | | | | 1.4 | | |  |
| F6UXX1 | Heterogeneous nuclear ribonucleoprotein Q (Fragment) | | | | 473.94 | | | | 68.11 | | | | 1 | | | 14 | | | 99 | | | | 1.3 | | |  |
| Q6ZU80-1 | Isoform 1 of Centrosomal protein of 128 kDa | | | | 10.33 | | | | 2.84 | | | | 2 | | | 3 | | | 3 | | | | 1.4 | | |  |
| O94986-1 | Isoform 1 of Centrosomal protein of 152 kDa | | | | 10.09 | | | | 1.80 | | | | 1 | | | 2 | | | 3 | | | | 1.4 | | |  |
| C9JRZ8-2 | Isoform 2 of Aldo-keto reductase family 1 member B15 | | | | 21.63 | | | | 6.96 | | | | 2 | | | 2 | | | 6 | | | | 1.4 | | |  |
| Q9NW15-2 | Isoform 2 of Anoctamin-10 | | | | 18.64 | | | | 10.85 | | | | 4 | | | 4 | | | 5 | | | | 1.4 | | |  |
| P53396-2 | Isoform 2 of ATP-citrate synthase | | | 1054.83 | | | | | | 56.28 | | | | | 1 | | 61 | | | | 278 | | | 1.4 | | |
| O43852-2 | Isoform 2 of Calumenin | | | | 129.38 | | | | 51.43 | | | | 2 | | | 14 | | | 30 | | | | 1.4 | | |  |
| Q86X95-2 | Isoform 2 of Corepressor interacting with RBPJ 1 | | | | 11.37 | | | | 12.87 | | | | 3 | | | 3 | | | 3 | | | | 1.3 | | |  |
| Q15375-2 | Isoform 2 of Ephrin type-A receptor 7 | | | | 13.04 | | | | 3.73 | | | | 1 | | | 3 | | | 4 | | | | 1.4 | | |  |
| Q9BSJ8-2 | Isoform 2 of Extended synaptotagmin-1 | | | | 386.29 | | | | 46.77 | | | | 36 | | | 38 | | | 94 | | | | 1.4 | | |  |
| Q8N2G8-2 | Isoform 2 of GH3 domain-containing protein | | | | 21.10 | | | | 9.49 | | | | 4 | | | 4 | | | 6 | | | | 1.4 | | |  |
| Q6F5E8-2 | Isoform 2 of Leucine-rich repeat-containing protein 16C | | | | 34.15 | | | | 5.47 | | | | 6 | | | 6 | | | 9 | | | | 1.8 | | |  |
| O00754-2 | Isoform 2 of Lysosomal alpha-mannosidase | | | | 55.62 | | | | 15.05 | | | | 11 | | | 11 | | | 14 | | | | 1.4 | | |  |
| Q15154-2 | Isoform 2 of Pericentriolar material 1 protein | | | | 143.40 | | | | 18.74 | | | | 1 | | | 29 | | | 36 | | | | 1.3 | | |  |
| Q9NRG1-2 | Isoform 2 of Phosphoribosyltransferase domain-containing protein 1 | | | | | 14.77 | | | | | 23.68 | | | 4 | | | 4 | | | 4 | | | | 1.4 | | |
| Q7L2E3-2 | Isoform 2 of Putative ATP-dependent RNA helicase DHX30 | | | | 556.88 | | | | 59.82 | | | | 2 | | | 59 | | | 142 | | | | 1.4 | | |  |
| Q9BVN2-2 | Isoform 2 of RUN and SH3 domain-containing protein 1 | | | | 12.21 | | | | 10.62 | | | | 3 | | | 3 | | | 3 | | | | 1.4 | | |  |
| O75157-2 | Isoform 2 of TSC22 domain family protein 2 | | | | 20.44 | | | | 4.23 | | | | 1 | | | 4 | | | 6 | | | | 1.4 | | |  |
| Q9ULD0-3 | Isoform 3 of 2-oxoglutarate dehydrogenase-like, mitochondrial | | | | 20.05 | | | | 5.37 | | | | 1 | | | 4 | | | 6 | | | | 1.4 | | |  |
| Q8WWI1-3 | Isoform 3 of LIM domain only protein 7 | | | | 60.30 | | | | 11.93 | | | | 11 | | | 11 | | | 16 | | | | 1.4 | | |  |
| Q7Z7H5-3 | Isoform 3 of Transmembrane emp24 domain-containing protein 4 | | | | | | 51.41 | | 34.41 | | | | 3 | | | 5 | | | 13 | | | | 1.4 | | |  |
| Q969Y2-3 | Isoform 3 of tRNA modification GTPase GTPBP3, mitochondrial | | | | 68.15 | | | | 28.24 | | | | 10 | | | 11 | | | 14 | | | | 1.5 | | |  |
| Q70UQ0-4 | Isoform 4 of Inhibitor of nuclear factor kappa-B kinase-interacting protein | | | | 92.57 | | | | 49.34 | | | | 13 | | | 17 | | | 25 | | | | 1.4 | | |  |
| Q9BXS6-4 | Isoform 4 of Nucleolar and spindle-associated protein 1 | | | | 20.34 | | | | 12.47 | | | | 5 | | | 5 | | | 6 | | | | 1.4 | | |  |
| Q6KCM7-6 | Isoform 6 of Calcium-binding mitochondrial carrier protein SCaMC-2 | | | | | | 11.48 | | 6.01 | | | | 1 | | | 1 | | | 2 | | | | 1.3 | | |  |
| O14662-2 | Isoform A of Syntaxin-16 | | | | 30.84 | | | | 12.50 | | | | 4 | | | 4 | | | 9 | | | | 1.3 | | |  |
| P50053 | Ketohexokinase | | | | 21.95 | | | | 23.83 | | | | 5 | | | 5 | | | 5 | | | | 1.8 | | |  |
| Q12840 | Kinesin heavy chain isoform 5A | | | | 114.12 | | | | 17.25 | | | | 5 | | | 15 | | | 29 | | | | 1.6 | | |  |
| O60282 | Kinesin heavy chain isoform 5C | | | | 122.56 | | | | 21.73 | | | | 4 | | | 19 | | | 32 | | | | 1.8 | | |  |
| O14777 | Kinetochore protein NDC80 homolog | | | | 85.53 | | | | 29.60 | | | | 15 | | | 15 | | | 19 | | | | 1.3 | | |  |
| Q9BRS8 | La-related protein 6 | | | | 11.74 | | | | 10.18 | | | | 3 | | | 3 | | | 3 | | | | 1.3 | | |  |
| Q9UN81 | LINE-1 retrotransposable element ORF1 protein | | | | 10.89 | | | | 9.17 | | | | 3 | | | 3 | | | 3 | | | | 1.4 | | |  |
| D6RCP8 | Lipoyl synthase, mitochondrial | | | | 12.95 | | | | 11.98 | | | | 2 | | | 2 | | | 3 | | | | 1.4 | | |  |
| P51608 | Methyl-CpG-binding protein 2 | | | | 47.16 | | | | 27.16 | | | | 9 | | | 9 | | | 12 | | | | 1.3 | | |  |
| Q9UNW1 | Multiple inositol polyphosphate phosphatase 1 | | | | 49.33 | | | | 27.52 | | | | 9 | | | 9 | | | 12 | | | | 1.4 | | |  |
| A0A087WUT0 | Myelin expression factor 2 | | | | 28.85 | | | | 13.89 | | | | 6 | | | 6 | | | 8 | | | | 1.5 | | |  |
| P35580 | Myosin-10 | 1400.71 | | | | | | | | | 57.79 | 109 | | | | | 137 | 343 | | | | | | 1.5 | | |
| Q96CV9 | Optineurin | | | | 10.62 | | | | 5.72 | | | | 3 | | | 3 | | | 3 | | | | 1.4 | | |  |
| Q8WYQ0 | Peroxin Pex6p | | | | 13.28 | | | | 7.18 | | | | 3 | | | 3 | | | 3 | | | | 1.4 | | |  |
| Q8NEB9 | Phosphatidylinositol 3-kinase catalytic subunit type 3 | | | | 90.81 | | | | 25.82 | | | | 17 | | | 17 | | | 25 | | | | 1.3 | | |  |
| Q9BTY2 | Plasma alpha-L-fucosidase | | | | 36.53 | | | | 17.34 | | | | 7 | | | 7 | | | 10 | | | | 1.3 | | |  |
| Q9UHG3 | Prenylcysteine oxidase 1 | | | | 86.53 | | | | 34.06 | | | | 13 | | | 13 | | | 20 | | | | 1.3 | | |  |
| Q8WUM4 | Programmed cell death 6-interacting protein | | | | 457.92 | | | | 63.82 | | | | 1 | | | 53 | | | 116 | | | | 1.4 | | |  |
| Q06323 | Proteasome activator complex subunit 1 | | | | 183.59 | | | | 68.27 | | | | 17 | | | 17 | | | 47 | | | | 1.6 | | |  |
| Q9UL46 | Proteasome activator complex subunit 2 | | | | 109.78 | | | | 52.30 | | | | 11 | | | 11 | | | 26 | | | | 1.4 | | |  |
| O94992 | Protein HEXIM1 | | | | 80.42 | | | | 33.15 | | | | 10 | | | 10 | | | 18 | | | | 1.4 | | |  |
| Q93096 | Protein tyrosine phosphatase type IVA 1 | | | | 24.34 | | | | 29.48 | | | | 2 | | | 4 | | | 6 | | | | 1.4 | | |  |
| Q7L2E3 | Putative ATP-dependent RNA helicase DHX30 | | | | 543.58 | | | | 59.46 | | | | 1 | | | 58 | | | 140 | | | | 1.3 | | |  |
| Q58FG1 | Putative heat shock protein HSP 90-alpha A4 | | | | 308.59 | | | | 16.99 | | | | 1 | | | 5 | | | 82 | | | | 3.9 | | |  |
| Q14964 | Ras-related protein Rab-39A | | | | 34.40 | | | | 16.59 | | | | 2 | | | 4 | | | 10 | | | | 1.4 | | |  |
| Q96DA2 | Ras-related protein Rab-39B | | | | 29.07 | | | | 9.39 | | | | 1 | | | 3 | | | 9 | | | | 1.6 | | |  |
| P20336 | Ras-related protein Rab-3A | | | | 46.51 | | | | 37.27 | | | | 1 | | | 7 | | | 14 | | | | 1.3 | | |  |
| O95716 | Ras-related protein Rab-3D | | | | 51.66 | | | | 36.99 | | | | 1 | | | 7 | | | 15 | | | | 1.4 | | |  |
| B4DTR1 | Receptor tyrosine-protein kinase erbB-2 | | | | 12.15 | | | | 3.58 | | | | 1 | | | 3 | | | 3 | | | | 1.4 | | |  |
| P35270 | Sepiapterin reductase | | | | 94.73 | | | | 43.68 | | | | 10 | | | 10 | | | 24 | | | | 1.6 | | |  |
| P49458 | Signal recognition particle 9 kDa protein | | | | 65.71 | | | | 50.00 | | | | 6 | | | 6 | | | 19 | | | | 1.4 | | |  |
| Q8TBP6 | Solute carrier family 25 member 40 | | | | 13.71 | | | | 16.57 | | | | 3 | | | 3 | | | 3 | | | | 1.3 | | |  |
| Q6SZW1 | Sterile alpha and TIR motif-containing protein 1 | | | | 90.12 | | | | 32.18 | | | | 16 | | | 16 | | | 22 | | | | 2.0 | | |  |
| O14656 | Torsin-1A | | | | 18.53 | | | | 18.67 | | | | 5 | | | 5 | | | 5 | | | | 1.4 | | |  |
| P05412 | Transcription factor AP-1 | | | | 25.65 | | | | 22.05 | | | | 4 | | | 5 | | | 7 | | | | 1.3 | | |  |
| A0A087WTC8 | Uncharacterized protein (Fragment) | | | | 14.61 | | | | 36.84 | | | | 1 | | | 1 | | | 2 | | | | 1.5 | | |  |
| Q9UBQ0 | Vacuolar protein sorting-associated protein 29 | | | | 96.30 | | | | 60.99 | | | | 1 | | | 9 | | | 22 | | | | 1.5 | | |  |
| P08670 | Vimentin | | 1596.60 | | | | | | 82.40 | | | | 57 | | | 62 | | | 408 | | | | 1.5 | | |  |
| Q96H79 | Zinc finger CCCH-type antiviral protein 1-like | | | | 76.53 | | | | 62.33 | | | | 13 | | | 13 | | | 19 | | | | 1.4 | | |  |
