# Supplementary material for: Proteomic Analysis of eIF5B Silencing-Modulated Proteostasis
Source: PLoS One. 2016 Dec 13;11(12):e0168387. doi: 10.1371/journal.pone.0168387 (PMC5154608; doi:10.1371/journal.pone.0168387)
Supplement: S3 Table — (DOCX) [file pone.0168387.s012.docx]

**S3 Table. The list of down-regulated proteins in eIF5B-KN1-293T cells compared to the control cells.**

| Accession | Description | | Score | | | | | Coverage (%) | | | Unique Peptides | Peptides | | PSMs | | eIF5B(-)/control | |
| --- | --- | --- | --- | --- | --- | --- | --- | --- | --- | --- | --- | --- | --- | --- | --- | --- | --- |
| O60613 | | 15 kDa selenoprotein | | | | 35.95 | | | 32.72 | | 5 | 5 | 9 | | 0.7 | |  |
| Q92747 | | Actin-related protein 2/3 complex subunit 1A | | | | 111.38 | | | 47.57 | | 13 | 14 | 28 | | 0.7 | |  |
| P84077 | | ADP-ribosylation factor 1 | | | | 307.40 | | | 67.40 | | 1 | 15 | 76 | | 0.6 | |  |
| Q9UJY4 | | ADP-ribosylation factor-binding protein GGA2 | | | | 23.73 | | | 17.62 | | 6 | 6 | 6 | | 0.7 | |  |
| P36404 | | ADP-ribosylation factor-like protein 2 | | | | 69.18 | | | 48.37 | | 8 | 8 | 17 | | 0.7 | |  |
| P10109 | | Adrenodoxin, mitochondrial | | | | 15.46 | | | 12.50 | | 3 | 3 | 5 | | 0.7 | |  |
| P06280 | | Alpha-galactosidase A | | | | 181.71 | | | 34.27 | | 13 | 13 | 44 | | 0.6 | |  |
| Q4VCS5 | | Angiomotin | | | | 377.13 | | | 46.77 | | 39 | 43 | 91 | | 0.5 | |  |
| P00966 | | Argininosuccinate synthase | | | | 25.37 | | | 20.87 | | 6 | 6 | 8 | | 0.7 | |  |
| E7ETZ4 | | Basic leucine zipper and W2 domain-containing protein 2 | | | | 119.07 | | | 41.18 | | 14 | 19 | 33 | | 0.7 | |  |
| Q6Y288 | | Beta-1,3-glucosyltransferase | | | | 13.28 | | | 10.64 | | 3 | 3 | 3 | | 0.7 | |  |
| P25098 | | Beta-adrenergic receptor kinase 1 | | | | 29.31 | | | 10.45 | | 6 | 6 | 8 | | 0.7 | |  |
| Q9NWK9 | | Box C/D snoRNA protein 1 | | | | 46.08 | | | 31.70 | | 12 | 12 | 13 | | 0.7 | |  |
| P54687 | | Branched-chain-amino-acid aminotransferase, cytosolic | | | | 63.67 | | | 32.90 | | 8 | 8 | 16 | | 0.6 | |  |
| P00918 | | Carbonic anhydrase 2 | | | | 368.53 | | | 71.54 | | 16 | 16 | 92 | | 0.4 | |  |
| P29373 | | Cellular retinoic acid-binding protein 2 | | | | 154.50 | | | 76.09 | | 10 | 10 | 37 | | 0.3 | |  |
| O43633 | | Charged multivesicular body protein 2a | | | | 56.46 | | | 23.87 | | 5 | 6 | 13 | | 0.7 | |  |
| Q9Y696 | | Chloride intracellular channel protein 4 | | | | 111.89 | | | 62.85 | | 12 | 12 | 26 | | 0.7 | |  |
| Q14019 | | Coactosin-like protein | | | | 58.71 | | | 65.49 | | 10 | 10 | 16 | | 0.6 | |  |
| Q14011 | | Cold-inducible RNA-binding protein | | | | 154.92 | | | 39.53 | | 6 | 6 | 35 | | 0.7 | |  |
| P83436 | | Conserved oligomeric Golgi complex subunit 7 | | | | 29.30 | | | 11.82 | | 6 | 6 | 7 | | 0.7 | |  |
| P17812 | | CTP synthase 1 | | | | 308.89 | | | 51.95 | | 28 | 32 | 78 | | 0.7 | |  |
| P53701 | | Cytochrome c-type heme lyase | | | | 56.42 | | | 38.06 | | 10 | 10 | 15 | | 0.7 | |  |
| O43175 | | D-3-phosphoglycerate dehydrogenase | | | | 720.88 | | | 63.98 | | 36 | 36 | 178 | | 0.7 | |  |
| P09884 | | DNA polymerase alpha catalytic subunit | | | | 199.46 | | | 30.71 | | 34 | 35 | 51 | | 0.7 | |  |
| Q9UBZ4 | | DNA-(apurinic or apyrimidinic site) lyase 2 | | | | 14.21 | | | 9.27 | | 3 | 3 | 4 | | 0.7 | |  |
| Q9Y2Y1 | | DNA-directed RNA polymerase III subunit RPC10 | | | | 27.35 | | | 60.19 | | 4 | 4 | 6 | | 0.7 | |  |
| Q99956 | | Dual specificity protein phosphatase 9 | | | | 133.30 | | | 41.41 | | 12 | 12 | 33 | | 0.7 | |  |
| Q05639 | | Elongation factor 1-alpha 2 | 1537.23 | | | | | | 65.87 | | 10 | 24 | 367 | | 0.6 | |  |
| A0A087WUT6 | | Eukaryotic translation initiation factor 5B | | | | 310.43 | | | 40.16 | | 50 | 50 | 83 | | 0.5 | |  |
| Q16658 | | Fascin | | | | 324.16 | | | 64.30 | | 25 | 25 | 76 | | 0.7 | |  |
| Q01469 | | Fatty acid-binding protein, epidermal | | | | 167.93 | | | 64.44 | | 13 | 13 | 40 | | 0.7 | |  |
| Q14192 | | Four and a half LIM domains protein 2 | | | | 66.09 | | | 43.01 | | 10 | 10 | 18 | | 0.7 | |  |
| P60520 | | Gamma-aminobutyric acid receptor-associated protein-like 2 | | | | 36.55 | | | 41.88 | | 6 | 6 | 11 | | 0.7 | |  |
| Q9NWZ8 | | Gem-associated protein 8 | | | | 11.81 | | | 7.02 | | 2 | 2 | 4 | | 0.7 | |  |
| P15104 | | Glutamine synthetase | | | | 80.44 | | | 37.53 | | 12 | 12 | 19 | | 0.7 | |  |
| A0A087WUQ6 | | Glutathione peroxidase 1 | | | | 49.50 | | | 61.88 | | 7 | 7 | 14 | | 0.5 | |  |
| Q9NPB8 | | Glycerophosphocholine phosphodiesterase GPCPD1 | | | | 35.36 | | | 16.52 | | 8 | 8 | 10 | | 0.7 | |  |
| P23434 | | Glycine cleavage system H protein, mitochondrial | | | | 56.29 | | | 17.34 | | 2 | 2 | 13 | | 0.7 | |  |
| Q7Z4Q2 | | HEAT repeat-containing protein 3 | | | | 80.78 | | | 21.91 | | 11 | 12 | 21 | | 0.7 | |  |
| P08107 | | Heat shock 70 kDa protein 1A/1B | 2707.46 | | | | | | 70.67 | | 31 | 55 | 651 | | 0.7 | |  |
| P09601 | | Heme oxygenase 1 | | | | 71.65 | | | 36.46 | | 9 | 9 | 17 | | 0.7 | |  |
| Q5JSL0 | | High mobility group nucleosome-binding domain-containing protein 5 | | | | 120.79 | | | 56.08 | | 1 | 8 | 31 | | 0.7 | |  |
| P17096 | | High mobility group protein HMG-I/HMG-Y | | | | 73.25 | | | 33.64 | | 2 | 5 | 16 | | 0.7 | |  |
| P16403 | | Histone H1.2 | | | | 246.26 | | | 39.44 | | 3 | 15 | 62 | | 0.6 | |  |
| P16402 | | Histone H1.3 | | | | 234.83 | | | 32.13 | | 1 | 13 | 59 | | 0.7 | |  |
| O60814 | | Histone H2B type 1-K | | 1295.01 | | | | | 73.02 | | 2 | 19 | 412 | | 0.6 | |  |
| P78318 | | Immunoglobulin-binding protein 1 | | | | 51.45 | | | 33.33 | | 8 | 8 | 14 | | 0.7 | |  |
| Q9BY32 | | Inosine triphosphate pyrophosphatase | | | | 141.02 | | | 77.32 | | 11 | 11 | 32 | | 0.7 | |  |
| Q96CN7 | | Isochorismatase domain-containing protein 1 | | | | 115.48 | | | 44.63 | | 11 | 11 | 28 | | 0.7 | |  |
| P49407-2 | | Isoform 1B of Beta-arrestin-1 | | | | 11.96 | | | 11.95 | | 3 | 3 | 3 | | 0.6 | |  |
| P62847-2 | | Isoform 2 of 40S ribosomal protein S24 | | | | 141.37 | | | 47.69 | | 1 | 8 | 39 | | 0.6 | |  |
| P08195-2 | | Isoform 2 of 4F2 cell-surface antigen heavy chain | | | | 313.71 | | | 48.39 | | 23 | 24 | 76 | | 0.5 | |  |
| Q9H857-2 | | Isoform 2 of 5'-nucleotidase domain-containing protein 2 | | | | 60.34 | | | 28.55 | | 11 | 11 | 16 | | 0.7 | |  |
| P08243-2 | | Isoform 2 of Asparagine synthetase [glutamine-hydrolyzing] | | | | 419.11 | | | 68.15 | | 31 | 32 | 107 | | 0.7 | |  |
| Q9Y597-2 | | Isoform 2 of BTB/POZ domain-containing protein KCTD3 | | | | 40.37 | | | 13.90 | | 5 | 7 | 9 | | 0.7 | |  |
| Q14444-2 | | Isoform 2 of Caprin-1 | | | | 236.73 | | | 32.71 | | 19 | 19 | 55 | | 0.7 | |  |
| Q96FN4-2 | | Isoform 2 of Copine-2 | | | | 24.04 | | | 7.40 | | 2 | 3 | 7 | | 0.6 | |  |
| Q5VTB9-3 | | Isoform 2 of E3 ubiquitin-protein ligase RNF220 | | | | 22.06 | | | 17.56 | | 5 | 5 | 6 | | 0.7 | |  |
| Q16576-2 | | Isoform 2 of Histone-binding protein RBBP7 | | | | 286.88 | | | 41.79 | | 1 | 15 | 66 | | 0.7 | |  |
| P51617-2 | | Isoform 2 of Interleukin-1 receptor-associated kinase 1 | | | | 70.30 | | | 20.67 | | 10 | 10 | 17 | | 0.6 | |  |
| Q9P2N7-2 | | Isoform 2 of Kelch-like protein 13 | | | | 20.99 | | | 9.79 | | 1 | 5 | 6 | | 0.6 | |  |
| Q16773-2 | | Isoform 2 of Kynurenine--oxoglutarate transaminase 1 | | | | 12.28 | | | 14.78 | | 3 | 3 | 3 | | 0.7 | |  |
| P84157-2 | | Isoform 2 of Matrix-remodeling-associated protein 7 | | | | 20.65 | | | 30.00 | | 4 | 4 | 5 | | 0.7 | |  |
| Q969Z3-2 | | Isoform 2 of Mitochondrial amidoxime reducing component 2 | | | | 19.16 | | | 15.56 | | 3 | 4 | 5 | | 0.7 | |  |
| Q14697-2 | | Isoform 2 of Neutral alpha-glucosidase AB | | | | 506.76 | | | 49.90 | | 2 | 38 | 125 | | 0.7 | |  |
| A6NJ78-2 | | Isoform 2 of Probable methyltransferase-like protein 15 | | | | 16.45 | | | 9.16 | | 2 | 2 | 4 | | 0.7 | |  |
| Q15052-2 | | Isoform 2 of Rho guanine nucleotide exchange factor 6 | | | | 18.93 | | | 7.56 | | 1 | 5 | 5 | | 0.7 | |  |
| Q96SI9-2 | | Isoform 2 of Spermatid perinuclear RNA-binding protein | | | | 250.16 | | | 55.93 | | 25 | 32 | 69 | | 0.7 | |  |
| P52788-2 | | Isoform 2 of Spermine synthase | | | | 78.36 | | | 49.52 | | 11 | 11 | 17 | | 0.7 | |  |
| Q9UQB8-3 | | Isoform 3 of Brain-specific angiogenesis inhibitor 1-associated protein 2 | | | | 123.27 | | | 57.81 | | 21 | 21 | 31 | | 0.7 | |  |
| Q14677-3 | | Isoform 3 of Clathrin interactor 1 | | | | 87.07 | | | 30.79 | | 1 | 15 | 23 | | 0.6 | |  |
| Q92796-3 | | Isoform 3 of Disks large homolog 3 | | | | 29.22 | | | 20.22 | | 6 | 6 | 7 | | 0.7 | |  |
| Q15054-3 | | Isoform 3 of DNA polymerase delta subunit 3 | | | | 72.51 | | | 40.56 | | 15 | 15 | 21 | | 0.7 | |  |
| Q8NG27-3 | | Isoform 3 of E3 ubiquitin-protein ligase Praja-1 | | | | 10.39 | | | 5.44 | | 2 | 2 | 3 | | 0.7 | |  |
| Q9Y6I3-3 | | Isoform 3 of Epsin-1 | | | | 51.67 | | | 24.00 | | 7 | 7 | 10 | | 0.7 | |  |
| P16591-3 | | Isoform 3 of Tyrosine-protein kinase Fer | | | | 28.14 | | | 20.53 | | 7 | 7 | 8 | | 0.7 | |  |
| Q16539-2 | | Isoform CSBP1 of Mitogen-activated protein kinase 14 | | | | 77.29 | | | 48.06 | | 2 | 13 | 20 | | 0.7 | |  |
| P36969-2 | | Isoform Cytoplasmic of Phospholipid hydroperoxide glutathione peroxidase | | | | 54.30 | | | 50.59 | | 8 | 8 | 14 | | 0.4 | |  |
| Q15056-2 | | Isoform Short of Eukaryotic translation initiation factor 4H | | | | 129.03 | | | 56.58 | | 1 | 13 | 37 | | 0.7 | |  |
| P10636-3 | | Isoform Tau-A of Microtubule-associated protein tau | | | | 16.95 | | | 11.71 | | 3 | 3 | 5 | | 0.7 | |  |
| Q01650 | | Large neutral amino acids transporter small subunit 1 | | | | 37.37 | | | 8.09 | | 5 | 5 | 10 | | 0.6 | |  |
| F5GXC7 | | L-lactate dehydrogenase A chain | | | | 570.83 | | | 88.89 | | 1 | 5 | 153 | | 0.7 | |  |
| P49137 | | MAP kinase-activated protein kinase 2 | | | | 47.45 | | | 19.00 | | 4 | 6 | 12 | | 0.7 | |  |
| O15479 | | Melanoma-associated antigen B2 | | | | 81.97 | | | 30.72 | | 11 | 11 | 22 | | 0.4 | |  |
| Q9Y4U1 | | Methylmalonic aciduria and homocystinuria type C protein | | | | 19.64 | | | 24.82 | | 5 | 5 | 6 | | 0.6 | |  |
| Q99797 | | Mitochondrial intermediate peptidase | | | | 69.89 | | | 26.37 | | 13 | 13 | 18 | | 0.7 | |  |
| P26038 | | Moesin | | | | 531.69 | | | 67.59 | | 32 | 46 | 138 | | 0.7 | |  |
| Q5JXX1 | | Mortality factor 4-like protein 2 (Fragment) | | | | 42.00 | | | 48.73 | | 7 | 7 | 11 | | 0.7 | |  |
| Q9H7C9 | | Mth938 domain-containing protein | | | | 27.55 | | | 44.26 | | 6 | 6 | 8 | | 0.7 | |  |
| P48681 | | Nestin | | | | 22.92 | | | 5.31 | | 6 | 6 | 6 | | 0.7 | |  |
| Q5T321 | | Neurobeachin | | | | 30.89 | | | 2.58 | | 2 | 6 | 9 | | 0.7 | |  |
| P07196 | | Neurofilament light polypeptide | | | | 82.44 | | | 25.41 | | 11 | 14 | 23 | | 0.6 | |  |
| E7ESP9 | | Neurofilament medium polypeptide | | | | 205.65 | | | 43.33 | | 27 | 30 | 52 | | 0.4 | |  |
| H7BZQ3 | | OTU domain-containing protein 5 (Fragment) | | | | 71.03 | | | 32.43 | | 8 | 8 | 15 | | 0.7 | |  |
| H7BZJ4 | | Peptidyl-prolyl cis-trans isomerase (Fragment) | | | | 16.36 | | | 43.28 | | 1 | 4 | 5 | | 0.7 | |  |
| Q13162 | | Peroxiredoxin-4 | | | | 343.27 | | | 64.21 | | 15 | 17 | 80 | | 0.7 | |  |
| Q16822 | | Phosphoenolpyruvate carboxykinase [GTP], mitochondrial | | | | 77.71 | | | 32.03 | | 14 | 14 | 19 | | 0.7 | |  |
| P00558 | | Phosphoglycerate kinase 1 | | | 1046.47 | | | | 90.65 | | 45 | 45 | 262 | | 0.7 | |  |
| O00625 | | Pirin | | | | 40.54 | | | 29.31 | | 6 | 6 | 10 | | 0.7 | |  |
| Q92613 | | Protein Jade-3 | | | | 11.47 | | | 4.13 | | 2 | 3 | 4 | | 0.7 | |  |
| P31949 | | Protein S100-A11 | | | | 92.84 | | | 46.67 | | 5 | 5 | 26 | | 0.7 | |  |
| Q99584 | | Protein S100-A13 | | | | 63.09 | | | 55.10 | | 7 | 7 | 17 | | 0.7 | |  |
| P98179 | | Putative RNA-binding protein 3 | | | | 74.83 | | | 21.02 | | 2 | 2 | 17 | | 0.7 | |  |
| P32322 | | Pyrroline-5-carboxylate reductase 1, mitochondrial | | | | 168.71 | | | 53.29 | | 11 | 12 | 37 | | 0.7 | |  |
| Q13283 | | Ras GTPase-activating protein-binding protein 1 | | | | 551.89 | | | 59.66 | | 20 | 21 | 125 | | 0.7 | |  |
| P51151 | | Ras-related protein Rab-9A | | | | 44.55 | | | 42.79 | | 5 | 5 | 11 | | 0.7 | |  |
| P23921 | | Ribonucleoside-diphosphate reductase large subunit | | | | 374.36 | | | 57.20 | | 35 | 35 | 91 | | 0.6 | |  |
| P60891 | | Ribose-phosphate pyrophosphokinase 1 | | | | 314.66 | | | 66.35 | | 14 | 25 | 76 | | 0.7 | |  |
| P11908 | | Ribose-phosphate pyrophosphokinase 2 | | | | 225.23 | | | 60.38 | | 9 | 20 | 54 | | 0.7 | |  |
| P51812 | | Ribosomal protein S6 kinase alpha-3 | | | | 124.05 | | | 35.27 | | 12 | 21 | 33 | | 0.7 | |  |
| Q9UNW9 | | RNA-binding protein Nova-2 | | | | 50.74 | | | 16.06 | | 2 | 6 | 14 | | 0.4 | |  |
| P17707 | | S-adenosylmethionine decarboxylase proenzyme | | | | 11.37 | | | 9.28 | | 3 | 3 | 3 | | 0.7 | |  |
| H0YE28 | | Selenoprotein H (Fragment) | | | | 16.44 | | | 32.98 | | 4 | 4 | 5 | | 0.6 | |  |
| Q9Y5X3 | | Sorting nexin-5 | | | | 90.04 | | | 40.59 | | 16 | 17 | 24 | | 0.7 | |  |
| Q8N0X7 | | Spartin | | | | 133.29 | | | 30.48 | | 16 | 16 | 32 | | 0.7 | |  |
| Q13309 | | S-phase kinase-associated protein 2 | | | | 30.56 | | | 19.58 | | 7 | 7 | 9 | | 0.7 | |  |
| Q8IX90 | | Spindle and kinetochore-associated protein 3 | | | | 55.37 | | | 24.76 | | 8 | 8 | 13 | | 0.7 | |  |
| P55809 | | Succinyl-CoA:3-ketoacid coenzyme A transferase 1, mitochondrial | | | | | 25.32 | | | 13.27 | 6 | 6 | 7 | | 0.5 | |  |
| H3BQX5 | | Sulfotransferase (Fragment) | | | | 27.92 | | | 24.71 | | 2 | 2 | 6 | | 0.4 | |  |
| P42285 | | Superkiller viralicidic activity 2-like 2 | | | | 284.89 | | | 47.50 | | 42 | 42 | 72 | | 0.7 | |  |
| Q8NFX7 | | Syntaxin-binding protein 6 | | | | 31.39 | | | 31.90 | | 6 | 6 | 8 | | 0.6 | |  |
| E9PJK1 | | Tetraspanin | | | | 45.90 | | | 35.76 | | 3 | 3 | 9 | | 0.6 | |  |
| Q96J01 | | THO complex subunit 3 | | | | 121.12 | | | 40.17 | | 13 | 13 | 31 | | 0.7 | |  |
| P62328 | | Thymosin beta-4 | | | | 10.76 | | | 50.00 | | 1 | 4 | 4 | | 0.6 | |  |
| P37837 | | Transaldolase | | | | 258.45 | | | 51.34 | | 22 | 22 | 74 | | 0.7 | |  |
| A2RQR6 | | Transcription elongation factor A (SII)-like 4 variant 1 | | | | 23.94 | | | 23.12 | | 4 | 4 | 7 | | 0.7 | |  |
| R4GND1 | | Ubiquitin-conjugating enzyme E2 E3 | | | | 39.35 | | | 36.00 | | 1 | 6 | 9 | | 0.7 | |  |
| P61960 | | Ubiquitin-fold modifier 1 | | | | 73.25 | | | 58.82 | | 2 | 2 | 13 | | 0.7 | |  |
| Q9BV40 | | Vesicle-associated membrane protein 8 | | | | 10.45 | | | 33.00 | | 3 | 3 | 3 | | 0.5 | |  |
